# Supplementary material for: COVRECON: automated integration of genome- and metabolome-scale network reconstruction and data-driven inverse modeling of metabolic interaction networks
Source: Bioinformatics. 2023 Jul 4;39(7):btad397. doi: 10.1093/bioinformatics/btad397 (PMC10356784; doi:10.1093/bioinformatics/btad397)

**COVRECON Matlab Toolbox**

COVRECON Toolbox applies a bio-mathematical methods to detect the metabolic interaction change between two phenotypes, combining a superpathway network construction and Inverse differential Jacobian methods.

**Requirements**

We recommend the following software for compatibility:

Matlab (tested on version 2022b)

Parallel Computing Toolbox (tested on version 7.7, optional)

Input Data:

Metabolomics data for two Phenotypes with the same data format as in Metabolomics_data_example.mat, where rows and lines are samples and metabolites respectively.

Transcriptomic data with the same data format as in T_data.csv (optional).

Statistic variable importance of metabolites with the same data format as in Metabolites_Importance.mat (optional).

**Workflow**

**1, Sim-Network**

Firstly, make sure your matlab running folding is the toolbox folder ‘COVRECON’, open the toolbox interface with COVRECON.m. It contains two main parts: a, Sim-network, to build a superpathway metabolic interaction network for selected metabolites; b, Inverse Jacobian, to calculate the differential Jacobian matrix.


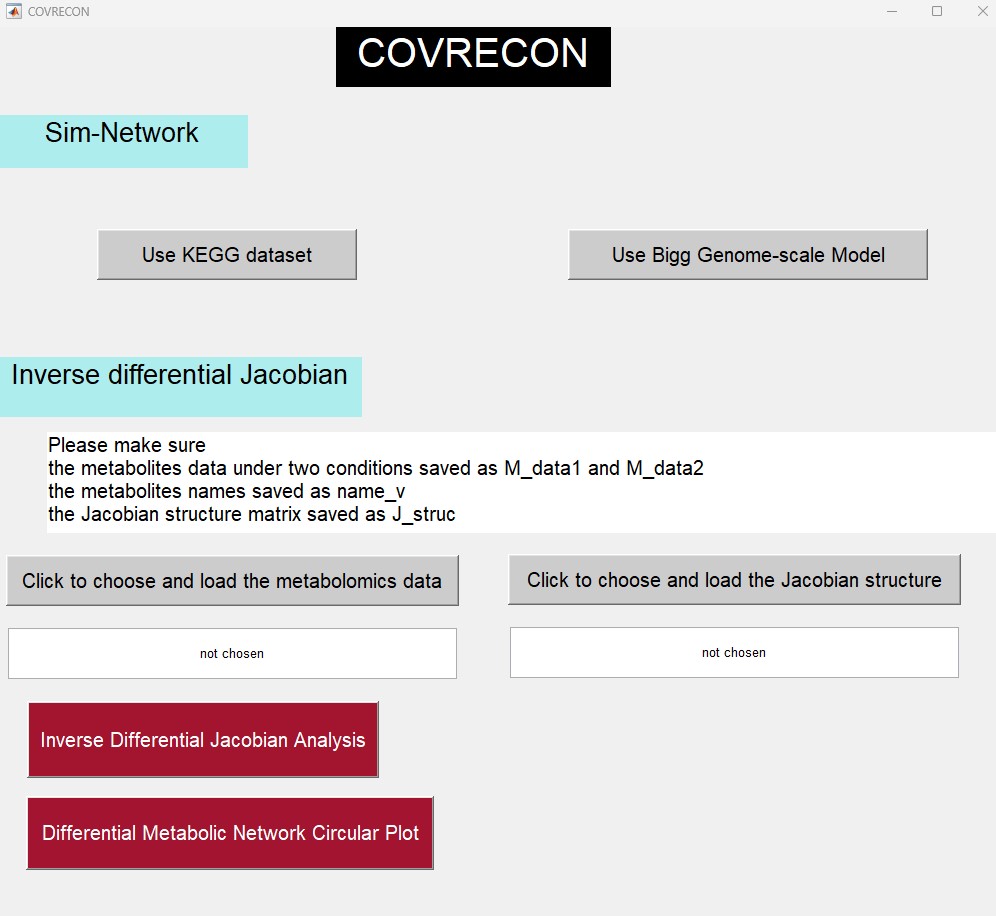


Choose to use KEGG database or use BIGG database. Here we put KEGG database as an example.

The KEGG Sim-network toolbox will be opened. The first two steps will build the organism specific database and generate the side-metabolites and selected-metabolites lists.


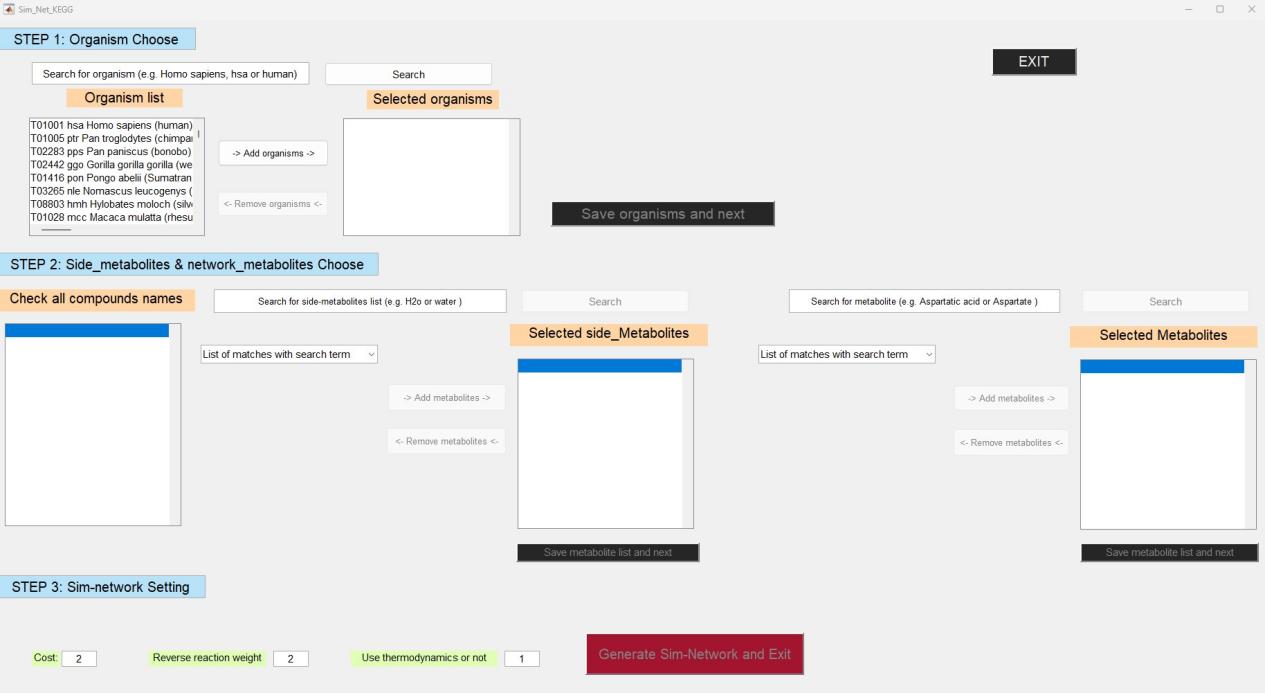


All KEGG organisms are list in the left box, each organism also includes information about alias and scientific classification for one to search.

The user can search for the species to include. Here it is possible for the user to include more than one organism if the samples of your data originate from different organisms. The user can always remove species from the selection list and to retrieve a list with all genomes the user selected. Once the user is happy with the selection, click the button ‘Save organisms and next’.

Now the toolbox will start to build the organism specific database. First, the software finds all enzymes (with E.C. numbers) associated with genes from the species by checking all KEGG reactions. A status bar shows the progress.


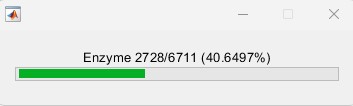


Second, the software finds all the reactions in the selected organism (non enzymatic reactions and enzymes included in the organism enzyme found in previous step). All metabolites in the reactions are also saved.


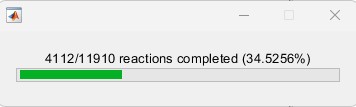


Last, the software loops through all compounds in KEGG and finds all their names and alias in KEGG.


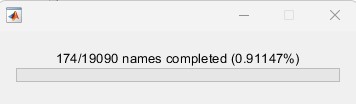


Theses steps will take several hours. Finally, the database has been built and saved in the folder ‘./database’. The organism specific database is kept unless one delete it and it will be automatically loaded next time when the same organism is chosen.

Following, all metabolites in the chosen organism will be listed in the left ‘check box’. Here, the user can search for side-metabolites to exclude and selected metabolites lists to build the network. Similarly, the user can always remove metabolites from the selection list and to retrieve a list. We have a pre-defined side-metabolites list. One need to select and decide the side-metabolites list and Selected-metabolites by click the ‘save ...and next’ button in turn.


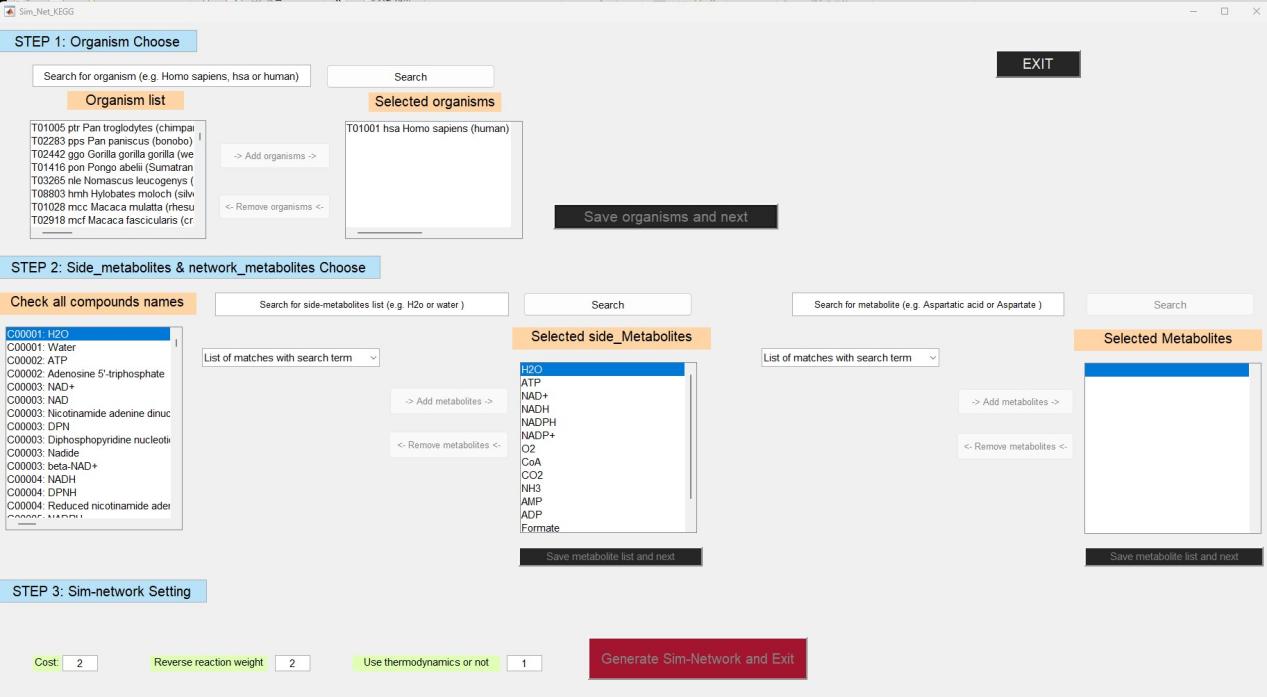

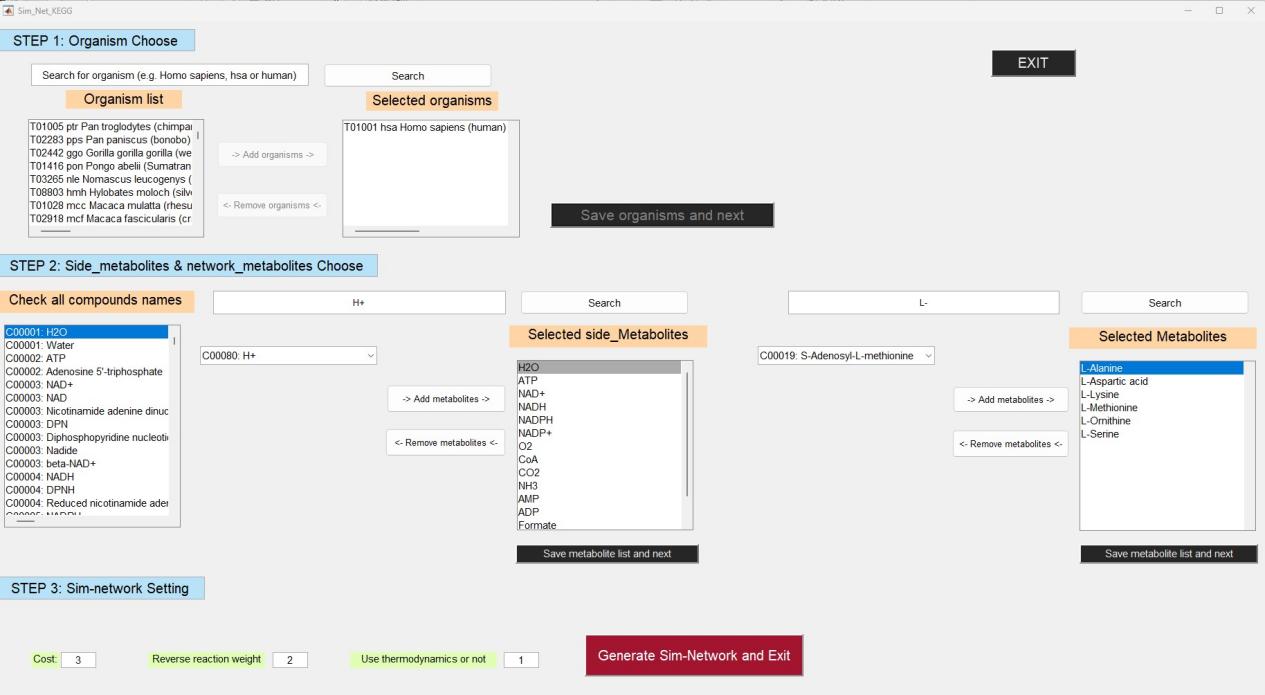


In the final STEP 3, the user need to set the settings and click the final red button to build the superpathway network for the selected metabolites.


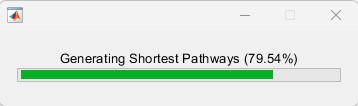


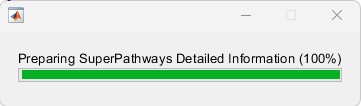


After the superpathways generation time, the Sim-Network interface will be closed and Jacobian structure and name list will be saved in the folder ‘./results/J_struc.mat’ and the SuperPathways information will be saved in ‘./results/path_r.mat’.,.

**2, Regression Loss based Inverse Jacobian Analysis**

Now, we are back to the COVRECON interface for the second step Inverse differential Jacobian analysis. Here we take the breast cancer analysis as a case study. The user can also run this part separately if the path information and Jacobian structure is already given and save in the same .mat format.

First, the user needs to click to choose and load the metabolomics data ./example/Metabolomics_data_example and the Jacobian structure data ./example/J_struc.mat.


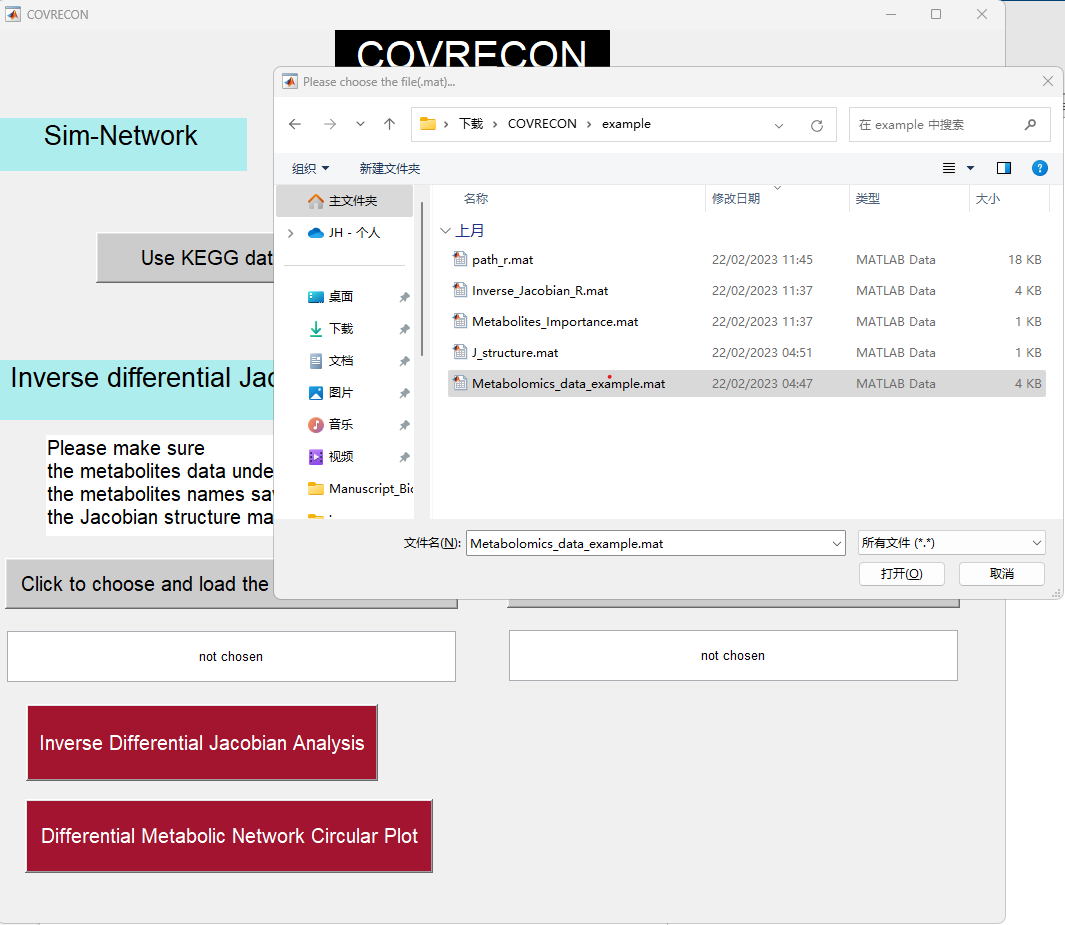


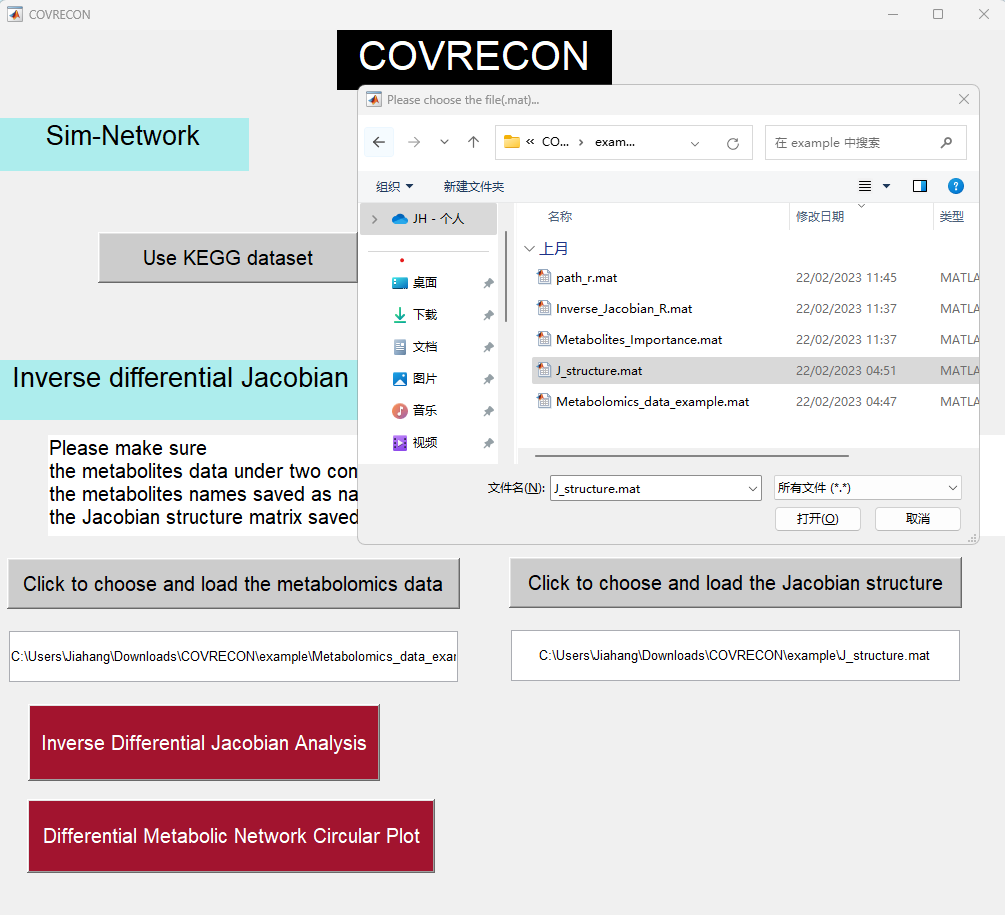


Then Click the red button ’Inverse Differential Jacobian Analysis’, after several minutes calculation, the resulted regression loss matrix R* will be saved in ‘./results/Inverse_Jacobian_R.mat’.

Meanwhile, a heatmap will be plotted with the following example format.


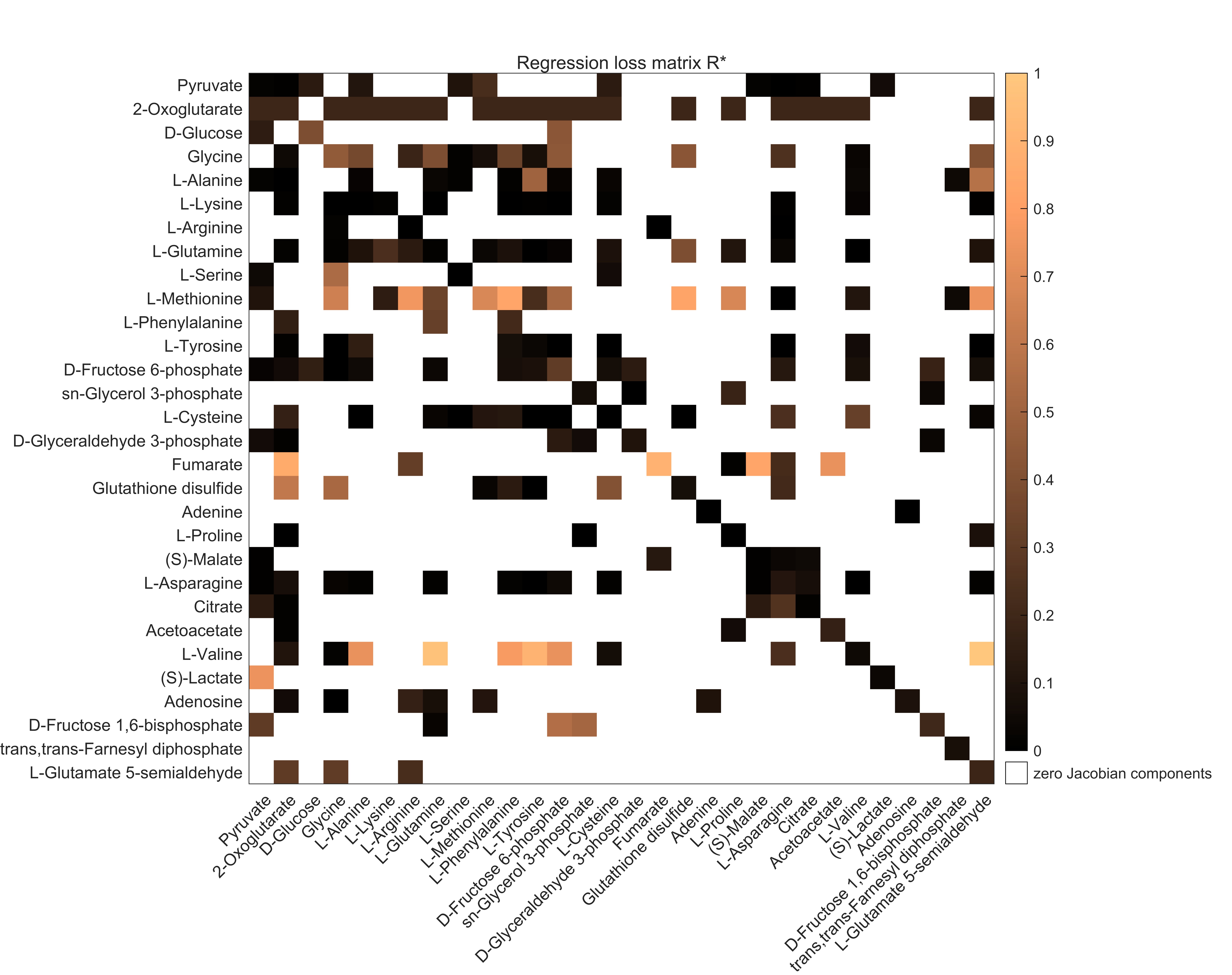


Finally, the user can do a circular plot for the Differential Metabolic Network. Click the red button ‘Differential Metabolic Network Circular Plot’, the circular-plot interface will pop up.


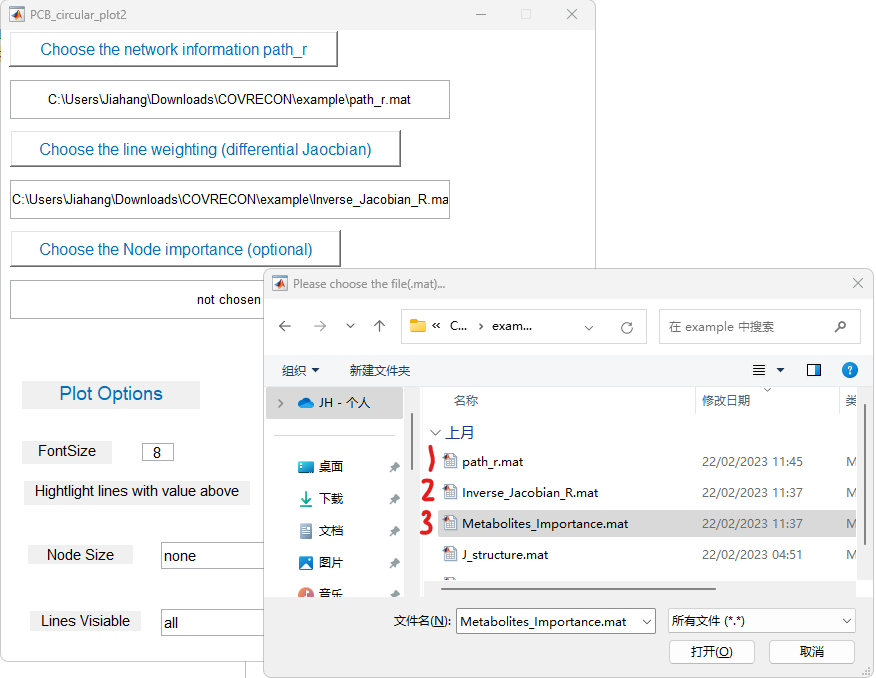


In the network graph plot, the user first need to upload three files first (example data in the folder ’example’): metabolic network results, path_r.mat; Inverse Differential Jacobian results, Inverse_Jacobian_R.mat; Metabolites Importance data, Metabolites_Importance.mat.


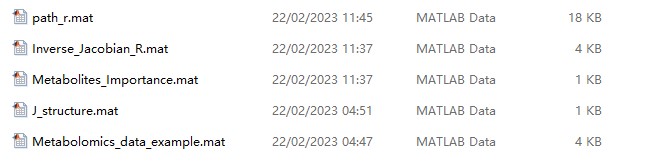


Following the user will have four options to plot the Differential Metabolic Network: FontSize for the metabolites names; Highlight Threshold for lines; Node Size and Lines Visible.


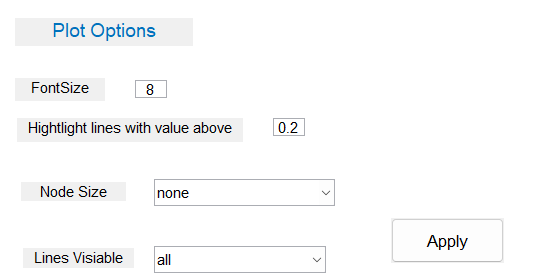


To take the Breast cancer data as an example. We first get the following graph using the default setting,


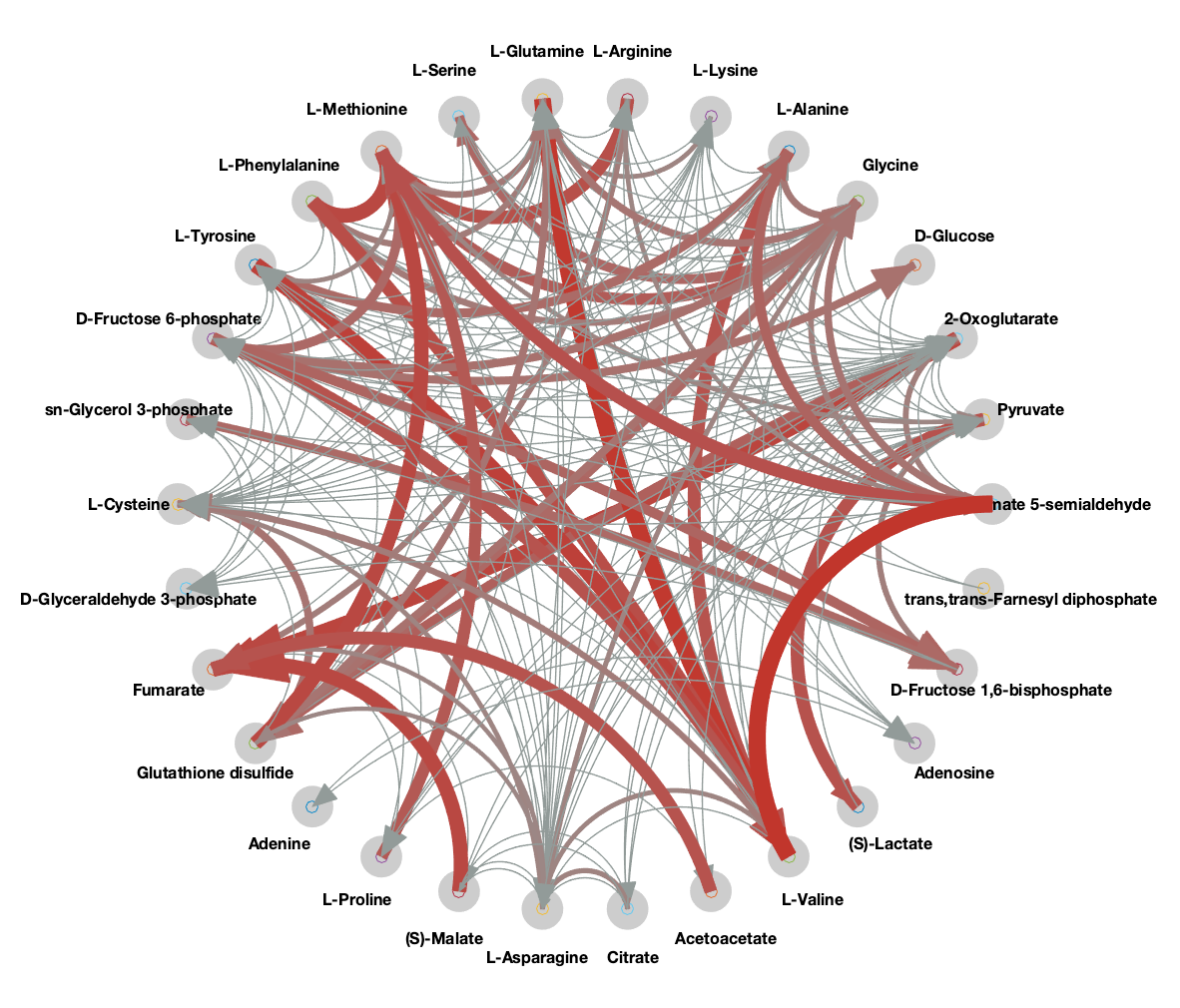


Then we change the settings as following: FontSize 12, line highlight threshold 0.5 and to use Metabolite Importance for the Node Size.


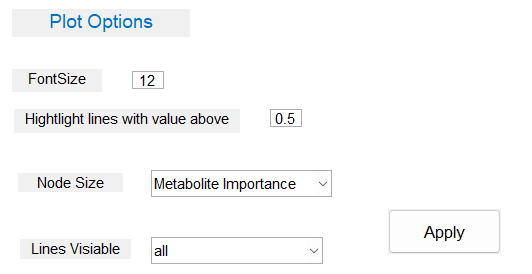


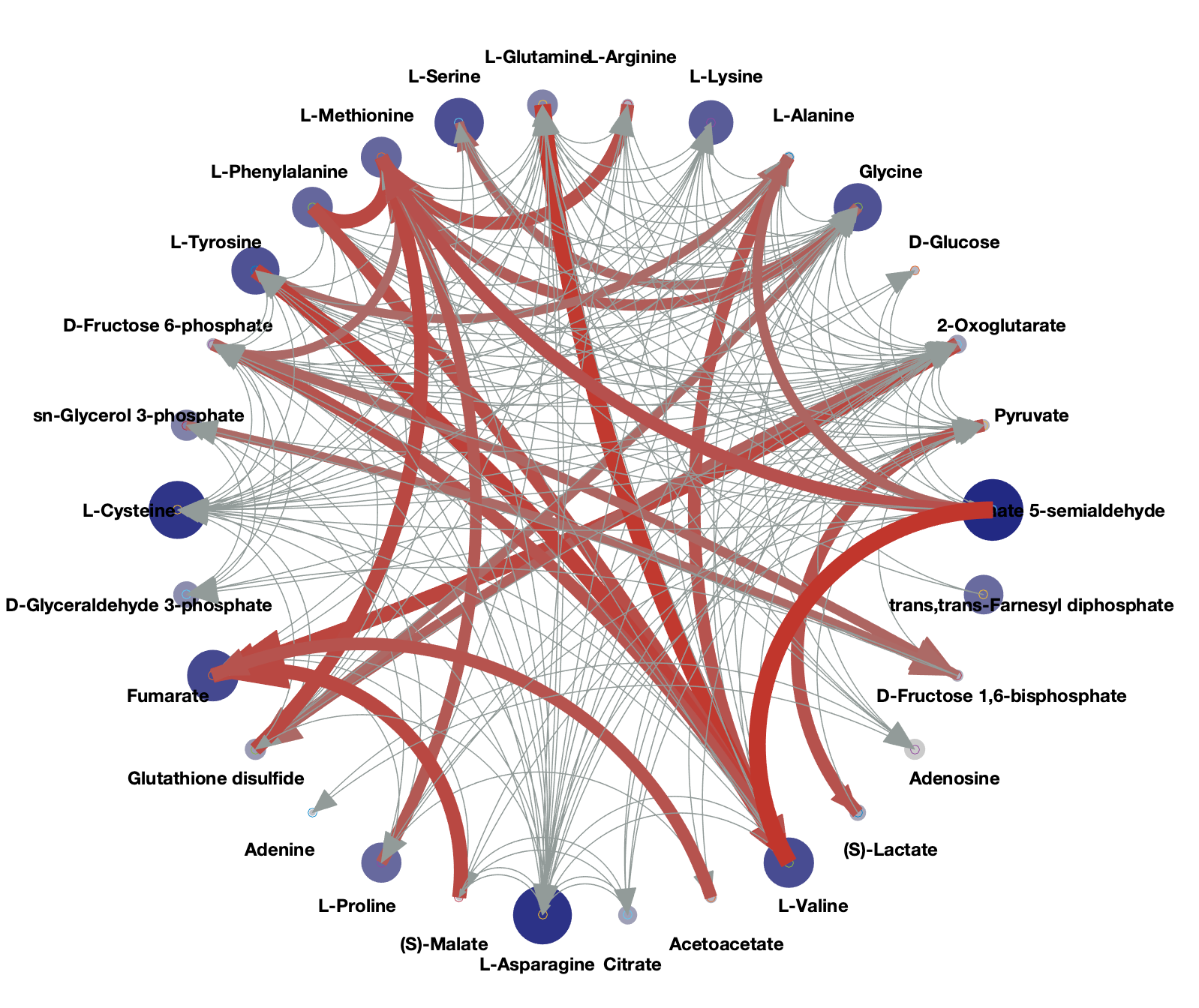


Finally, in order to make the network clearer, change the finally option Line Visible to only highlighted lines.


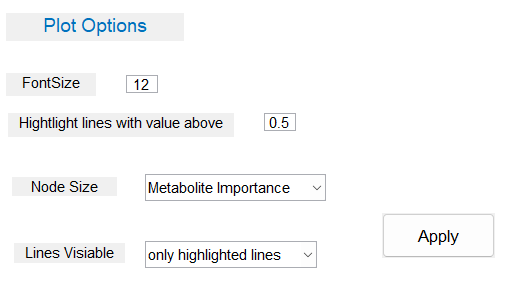


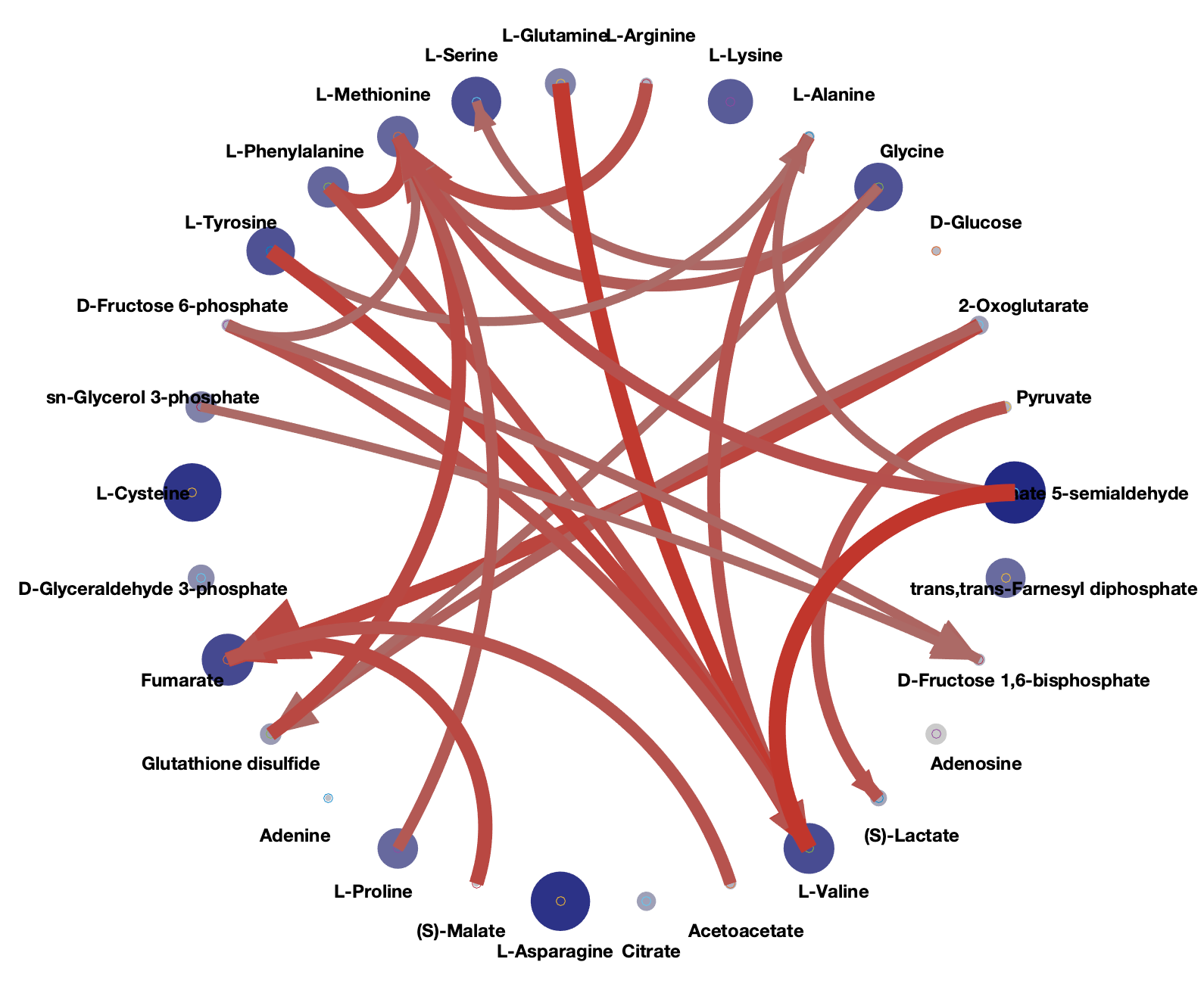


**Detailed Information for the Differential Metabolic Network**

In the Differential Metabolic Network, the user can use the matlab dataframe to chick and check the details of the metabolites and metabolic interactions. For metabolic information, detailed metabolites paths of the superpathways , reactions involved, Enzymes and organism genes will be listed.


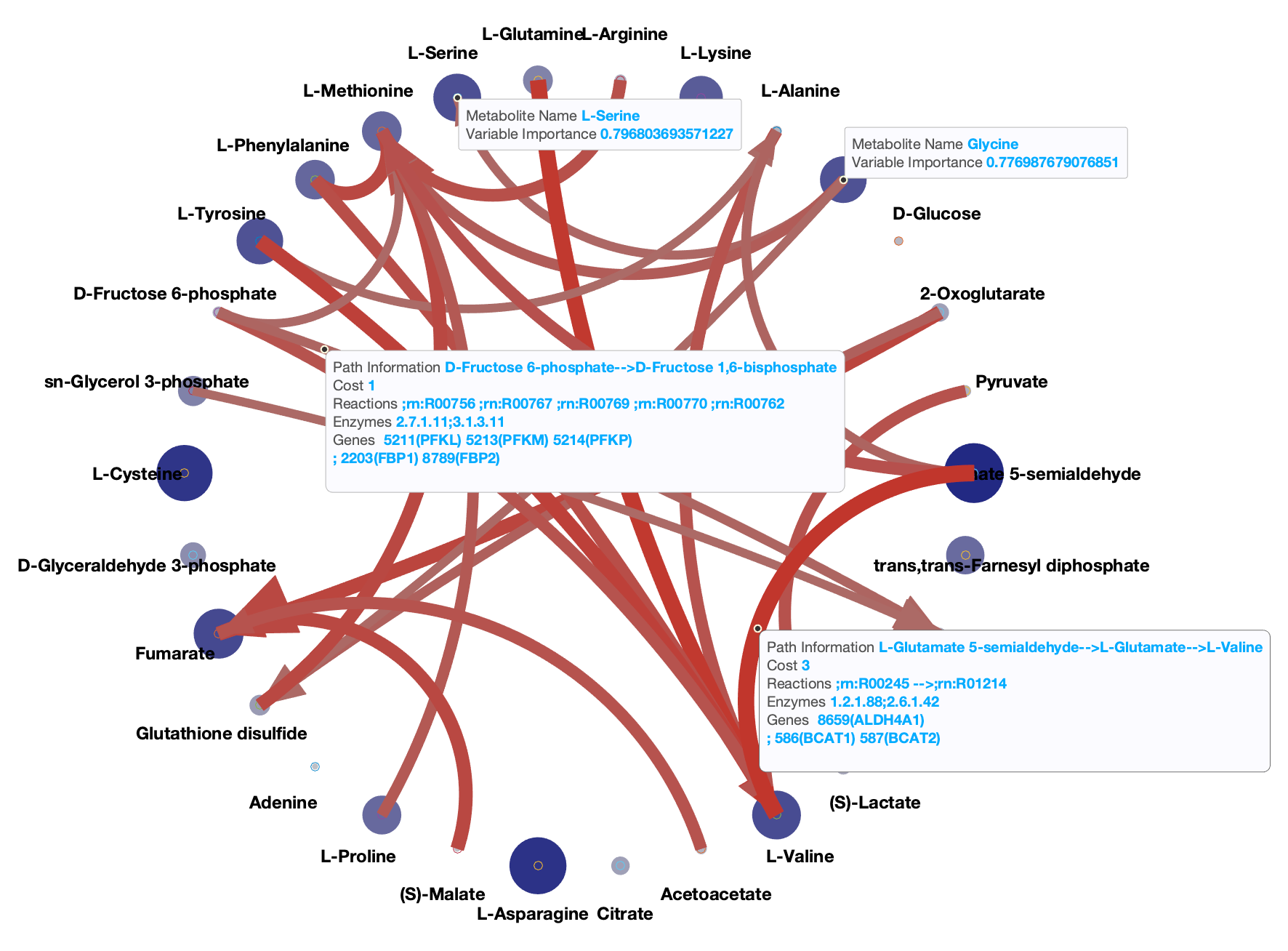

Supplement: btad397_Supplementary_Data [file btad397_supplementary_data.zip › Supplemental material S3 Toolbox Manual.docx]
