# Supplementary material for: COVRECON: automated integration of genome- and metabolome-scale network reconstruction and data-driven inverse modeling of metabolic interaction networks
Source: Bioinformatics. 2023 Jul 4;39(7):btad397. doi: 10.1093/bioinformatics/btad397 (PMC10356784; doi:10.1093/bioinformatics/btad397)
Supplement: btad397_Supplementary_Data [file btad397_supplementary_data.zip › Supplementary material S1.docx]

Supplemental Material

Inhalt

[1 Regression loss Jacobian algorithm 2](#_Toc1066458858)

[2 L-p optimization approach 5](#_Toc1075508544)

[3 Six components test model and all evaluation models 7](#_Toc702242209)

[4 Evaluation approach 9](#_Toc14682751)

[5 Effect of the network reduction on the Jacobian reconstruction 11](#_Toc1959860299)

[6 Sim-Network Matlab Interface and default settings 13](#_Toc1267867607)

[7 Supplementary figures and tables 15](#_Toc1718125315)

[Supplementary Figure 1. 15](#_Toc1467051643)

[Supplementary Table 1. 16](#_Toc1477212694)

[Supplementary Figure 2 17](#_Toc455305091)

[Supplementary Figure 3. 19](#_Toc828430176)

[Supplementary Figure 4 20](#_Toc1289484531)

[Supplementary Figure 5 21](#_Toc2109030640)

[Supplementary Figure 6 22](#_Toc1099009785)

[Supplementary Figure 7 23](#_Toc550608648)

[Supplementary Figure 8 24](#_Toc572512013)

# 1 Regression loss Jacobian algorithm

In this section, we will present the details of the regression loss Jacobian algorithm. We first start with the preliminary below:

**Preliminary: when** $\boldsymbol{K}(A)$ **is large, the regression loss is more stable than linear regression solution.**

In the inverse problem, matrix A consists of Covariance matrix components. It is constant during the inverse approach. Thus, we analyze the stability by giving a perturbation $\alpha/2$ to B, $B_{1}=B.* (1\pm\alpha/2*randB)$, $randB$ is a random matrix with the same size of B, in the value scope (0, 1). Assume the regression solution and loss of $A*x=B_{1}$ are ${x^{*}}_{1}$ and $r1$. Thus based on poor conditional linear equation solution analysis [1, 2], we know that:

$${max}_{B_{1}}\left\| \frac{{{x^{*}}_{1}-x}^{*}}{x^{*}} \right\|={max}_{B_{1}}\boldsymbol{(K}\left( A \right)\left\| \frac{B_{1}-B}{B} \right\|)=\boldsymbol{K}\left( A \right)*\alpha/2$$

(S1)

In equation (S1), we obtain the fluctuation scope of solution $x^{*}$ induced from perturbation of B. It implies that the fluctuation of $x^{*}$ is rather large when $\boldsymbol{K}\left( A \right)$ is large. On the other hand, for the regression loss,

$$\left\| r1-r \right\|=\left\| B_{1}-B-A*{x^{*}}_{1}+A*x^{*} \right\|\leq\left\| B_{1}-B \right\|+\left\| A*{x^{*}}_{1}-A*x^{*} \right\|=\left\| B_{1}-B \right\|+\left\| A*{{(A}^{T}A)}^{-1}*A^{T}B_{1}-A*{{(A}^{T}A)}^{-1}*A^{T}B \right\|\leq2\left\| B_{1}-B \right\|\leq\alpha\left\| B \right\|$$

We know that regression $r\propto\left\| B \right\|$, assume $r=\rho*\left\| B \right\|$, thus,

$$\frac{r1}{r}\in[\frac{\rho-\alpha}{\rho},\frac{\rho+\alpha}{\rho}]$$

(S2)

From Eq. (S2), we conclude that when $\alpha<\rho$ regression loss is perturbed within$[\frac{\rho-\alpha}{\rho},\frac{\rho+\alpha}{\rho}]$ for small perturbations $\alpha$ in B. Since in the inverse Jacobian algorithm, the non-diagonal components in D are ignored, inducing the errors in B and large regression loss values. Thus, $\rho is large, not close to 0$, which makes regression loss $r$ stable within a small scope $[\frac{\rho-\alpha}{\rho},\frac{\rho+\alpha}{\rho}]$.

To illustrate this linear equation solution instability, we make a simple example as below:

$A=\left( \begin{matrix} \begin{matrix} 1 & 100 \\ 0 & 1 \end{matrix} & \begin{matrix} 1 & 1 \\ 0 & 0 \end{matrix} \\ \begin{matrix} 1 & 0 \\ \begin{matrix} 0 \\ \begin{matrix} 2 \\ \begin{matrix} 0 \\ 0 \\ 1 \end{matrix} \end{matrix} \end{matrix} & \begin{matrix} 0 \\ \begin{matrix} 0 \\ \begin{matrix} 1 \\ 1 \\ 1 \end{matrix} \end{matrix} \end{matrix} \end{matrix} & \begin{matrix} \begin{matrix} 2 \\ \begin{matrix} 2 \\ 1 \end{matrix} \end{matrix} & \begin{matrix} 0 \\ \begin{matrix} 1 \\ 0 \end{matrix} \end{matrix} \\ \begin{matrix} 2 \\ 0 \\ 0 \end{matrix} & \begin{matrix} 0 \\ 2 \\ 0 \end{matrix} \end{matrix} \end{matrix} \right),$ $B=\left( \begin{matrix} \begin{matrix} \begin{matrix} 1 \\ 0 \\ 1 \end{matrix} \\ 0 \end{matrix} \\ \begin{matrix} 0 \\ \begin{matrix} 1 \\ 1 \\ 0 \end{matrix} \end{matrix} \end{matrix} \right)$, $\alpha=0.2, K (A)\sim100$

Now we calculate the solution and regression loss fluctuations ${|1-{{x^{*}}_{1}. /x}^{*}|}_{0}$ and $\left| 1-r1/r \right|$. Here ${|X|}_{0}$is the L-0 norm of X. In the 100 repeats of the random perturbation $\alpha$ and linear regression calculation, the calculated mean $\left| 1-r1/r \right|$ is 0.095, but the mean ${|1-{{x^{*}}_{1}. /x}^{*}|}_{0}$is 11.92. This test case shows the regression loss $r$ is much more stable compared to the regression solution $x^{*}$, even when matrix number $\boldsymbol{K}(A)\sim100$. And actually the number $\boldsymbol{K}(A)$ will mostly be larger than ${10}^{5}$ in a metabolic model (refer to the evaluation models overviews in Table 1), which implies a larger instability of linear regression solution $x^{*}$.

Since our aim is to analyze the large values in differential Jacobian matrix $D\boldsymbol{J}$ in Eq.4, the previous methods works through calculate the value of both Jacobian matrix $\boldsymbol{J}_{\boldsymbol{h}}$ and $\boldsymbol{J}_{\boldsymbol{d}}$with linear regression [1, 3-5]. This approach involves the ill-conditional linear regression problem, which makes them unstable. On the other hand, from the **Preliminary** we know that the regression loss is much more stable in the linear regression approach. In addition, our aim is to find the differential components between the two conditional Jacobian matrixes, the exact values of the difference are not important. In this work, utilizing the regression loss, we developed a new algorithm focusing on only finding the differential components in the two conditional Jacobian matrixes without calculating the exact values. This algorithm works through calculating the regression loss matrix $R^{*}=[r^{*}(I)]$ to represent the importance of differential Jacobian matrix components.

**Regression loss based inverse Jacobian algorithm**

Firstly, the regression loss matrix $R^{*}$ has the same matrix structure with both Jacobian matrix $\boldsymbol{J}_{\boldsymbol{h}}$ and $\boldsymbol{J}_{\boldsymbol{d}}$. But we change the zeros to *NaN* in $R^{*}$. For the others, we calculate a representative value $r^{*}(I)$ for every non-zero Jacobian component (identity$I\in\{1,2\ldots L\}$). From the hypothesis, we know that only few components in $q_{h}$ and $q_{d}$ are different. Here, for each $I$, we assume that the different component in $q_{h}$ and $q_{d}$ is only one, $I_{diff}=\{I\}$; the other (L-$1$) components$I_{same}=\left\{ 1,2\ldots L \right\}-I_{diff}$ are the same: $q_{h}\left( I_{same} \right)=q_{d}\left( I_{same} \right)\underset{\to}{\Delta}q_{same}$. Thus, one can combine the two equations in two conditions as following (refer to equation Eq.S5):

$$A_{combine}*{[q}_{h, diff};q_{d, diff};q_{same}]=[b_{h};b_{d}]$$

$$(S3)$$

In which, $A_{combine}$ is calculated from $A_{h}$ and $A_{d}$； $q_{combine}{=[q}_{h, diff};q_{d, diff};q_{same}]$ is the vector of unknown variables and $b_{combine}=[b_{h};b_{d}]$ is the vector composed of the two theoretical fluctuation matrixes values.

We can see that$A_{combine}$, $q_{combine}$ and $b_{combine}$ complies with the linear relationship, However, in biological experiments or in silicon simulations, the real covariance matrix or related $A_{combine}$ is not perfect linear against the theoretical perturbations $b_{combine}$, for the sample size or replicates are not infinite. We denote the real $b_{combine}^{r}$ as the sum of theoretical perturbations $b_{combine}$ and errors $\varepsilon_{b}=\varepsilon_{D}*rand (b)$, $b_{combine}^{r}=b_{combine}+\varepsilon_{b}$. Here, $rand (b)$ is a random matrix with the same size of b.

Together with the structure information of $b_{combine}$, we can now calculate the representative regression loss $r^{*}(I)$ for the differential components $I_{diff}=\{I\}$ as an optimization problem. In this optimization approach, we will do the linear regression between $A_{combine}(I, s)$ and $b_{combine}^{s}(I,b_{combine})$, by setting $I\in\{1,2\ldots L\}$ is the identity of the only differential Jacobian component $I_{diff}$, and $b_{combine}^{s}$ is from a set of random generated perturbation vectors with the matrix structure of $b_{combine}$, $S=\{ b_{combine}^{s}, s=1,2\ldots,t\}$. Thus, the linear regression cost $r (I, s)$ is a function of the chosen component $I$ and the sampled $b_{combine}^{s}$. In which, the influence of $s$ originates from the $\varepsilon_{b}$, and the influence of $I$ is the mistake loss from the wrong suggestion of $I$.

Assume the number of real differential Jacobian components is $k^{*}$, we can use the following optimization approach to plot the regression loss matrix $R^{*}=[r^{*}(I)]$ and find all the differential Jacobian components as the top$k^{*}$ $minimum-$valued components of the following regression cost $r^{*}(I)$:

$$r^{*} \left( I \right)=\min_{s} \left( r \left( I, s \right) \right)$$

$$(S4)$$

We put the inverse algorithm as below:

$$step1:randomly generate t perturbation vector samples set S=\{ b_{combine}^{s} \}with the$$

$$same tructure of b_{combine}.$$

$$step2:for every I= 1,2,\ldots,L, calculate A_{combine}\left( I, s \right).$$

$$step3:for every I, calculate regression loss r \left( I, s \right), and calculate r^{*} \left( I \right)=$$

$$\min_{s} \left( r \left( I, s \right) \right). Nornalize all the loss value to \left[ 0,1 \right], draw the regression loss matrix R^{*}=[r^{*}(I)].$$

$$step4:find the top k^{*} min{imum r}^{*} \left( I \right), I_{diff}={\arg k^{*} min}_{I}(r^{*} \left( I \right))$$

$$({S4}^{*})$$

Here, the regression loss is L2 cost; the set $S=\{ b_{combine}^{s} \}$ is randomly sampled from even distribution in (0, 1) with respect to the matrix structure $b_{combine}$. The sampling set S is applied to discriminate the influence of *b*, further the generated $r^{*}(I)$ represents the influence of $I$. One can plot the regression loss matrix $R^{*}=[r^{*}(I)]$ in $step3$ to represent the calculated differential Jacobian matrix. Also one can find $k^{*}$ differential Jacobian components as in the top min $k^{*}$ components in $R^{*}=[r^{*}(I)]$.

# 2 L-p optimization approach

In this section, we presents the details of the L-p optimization and an improved one.

Suppose in Eq. (3), Covariance matrix C is known, we rewrite the original equation into the linear equations with Jacobian matrix as unknown vector $X$,

$$A*X=b, A\in R\left( \frac{n\cdot\left( n+1 \right)}{2}, n^{2} \right), X\in R\left( n^{2},1 \right), b\in R\left( \frac{n\cdot\left( n+1 \right)}{2},1 \right)$$

(S5)

In which, X is all the independent variables in Jacobian matrix ***J***, it has the size of $\left( n^{2},1 \right)$; matrix A is the linear sum of the variables in covariance matrix C, it has the size of $\left( \frac{n\cdot\left( n+1 \right)}{2},1 \right)$; matrix b is all the independent variables in Fluctuation matrix D, it has the size of $\left( \frac{n\cdot\left( n+1 \right)}{2},1 \right)$. On the other hand, the Jacobian structure information will determine some variables in the Jacobian matrix to 0. Assume the Jacobian matrix contains $m$ non-zero variables.

$A*X=0 \to\left[ A_{final} A_{zeros} \right]\left[ \begin{matrix} x_{final} \\ x_{zeros} \end{matrix} \right]= b \to A_{final}*x_{final}=b,$ $A_{final}\in R\left( \frac{n\cdot\left( n+1 \right)}{2}, m \right)$, $x_{final}\in R\left( m,1 \right)$

(S6)

$$then x can be solved from the optimization problem of$$

$$\left\| A_{final}\hat{x}-b \right\|_{2}=min\left\{ \left\| A_{final}x-b \right\|_{2}|x\in R\left( m,1 \right) \right\}$$

(S7)

The problem state upon in Eq. (S7) is under the assumption that fluctuation matrix D is determined. However, D is unknown. Thus, the problem is solved using L-p optimization [5], as following:

$$b_{h}, b_{d}=\min\left\| \frac{{x^{'}}_{h}-{x^{'}}_{d}}{{x^{'}}_{h}} \right\|_{p,\varepsilon}^{p},$$

$${x^{'}}_{h}=\min\left\| A_{final,h}x-b_{h} \right\|_{2}, {x^{'}}_{h}\in R\left( m,1 \right)$$

$${x^{'}}_{d}=\min\left\| A_{final,d}x-b_{d} \right\|_{2}, {x^{'}}_{h}\in R\left( m,1 \right)$$

(S8)

in which, ‘h’ and ‘d’ represent the data condition ‘healthy’ and ‘diseased’, respectively. Value $\left\| X \right\|_{p,\varepsilon}^{p}$ is the p-norm of X, 0<p<1.

Finally the differential Jacobian DJ is calculated as:

$${D\boldsymbol{J}}_{ij}=\left\{ \begin{aligned} \left| \frac{{\boldsymbol{(}\boldsymbol{J}_{\boldsymbol{d}}\boldsymbol{)}}_{\boldsymbol{ij}}}{{\boldsymbol{(J}_{\boldsymbol{h}}\boldsymbol{)}}_{\boldsymbol{ij}}} \right| \\ 1, {\boldsymbol{if (J}_{\boldsymbol{h}}\boldsymbol{)}}_{\boldsymbol{ij}}\boldsymbol{=0.} \end{aligned} \right.$$

(S9)

here, $\boldsymbol{J}_{\boldsymbol{d}}$ and $\boldsymbol{J}_{\boldsymbol{h}}$ are assembled from the relevant values in ${x^{'}}_{h}$ and ${x^{'}}_{d}$.

This optimization approach is valid based on the assumption that only few elements will change largely under two conditions, ‘health’ and ‘disease’ conditions [5].

In the previous L-p optimization Jacobian algorithm, D is assumed to be a diagonal matrix. However, in real practice, the non-diagonal part will also make an influence. To that end, we include the non-diagonal part of D matrix into optimization. For this improved L-p Jacobian optimization with more parameters, we combine three optimization-methods: Particle-swarm search (PSO), Genetic Algorithm, and Pattern Search as one optimization step, where for every optimization step, half of the initial values are sampled near the best result in last optimization step, and the other half is randomly sampled within the search scope. This search is iterated until the improved value of L-p cost over 3 optimization steps is less than a preset threshold. Moreover, this workflow is repeated 20 times, each yielding one optimized result for the differential Jacobian, while the final differential Jacobian is computed as the average of five lowest L-p loss results.


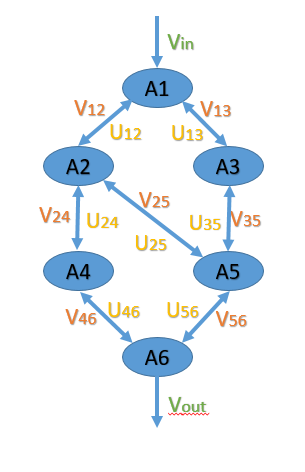
3 Six components test model and all evaluation models
To evaluate different inverse Jacobian methods, we constructed a six-compounds network,

Figure S1. Structure figure of test model.

the scheme figure of this network is shown in figure S1. The six compounds are denoted as$A_{i}$, with the concentration |$A_{i}$|. All including reactions in this network are listed in Eq. (S9). To simplify the problem, every reaction is assumed reversible mass action-reaction. The exact Jacobian matrixes with two different parameters are shown in Eq. (S10), representing the biological system under health and disease conditions, respectively. Where three Jacobian components are enhanced for the disease condition.

$$\frac{d\left| A_{1} \right|}{dt}=f1=v_{in}-v_{12}\cdot\left| A_{1} \right|+u_{12}\cdot\left| A_{2} \right|-v_{13}\cdot\left| A_{1} \right|+u_{13}\cdot\left| A_{3} \right|$$

$$\frac{d\left| A_{2} \right|}{dt}=f2=v_{12}\cdot\left| A_{1} \right|-u_{12}\cdot\left| A_{2} \right|-v_{24}\cdot\left| A_{2} \right|+u_{24}\cdot\left| A_{4} \right|-v_{25}\cdot\left| A_{2} \right|+u_{25}\cdot\left| A_{5} \right|$$

$$\frac{d\left| A_{3} \right|}{dt}=f3=v_{13}\cdot\left| A_{1} \right|-u_{13}\cdot\left| A_{3} \right|-v_{35}\cdot\left| A_{3} \right|+u_{35}\cdot\left| A_{5} \right|$$

$$\frac{d\left| A_{4} \right|}{dt}=f4=v_{24}\cdot\left| A_{2} \right|-u_{24}\cdot\left| A_{4} \right|-v_{46}\cdot\left| A_{4} \right|+u_{46}\cdot\left| A_{6} \right|$$

$$\frac{d\left| A_{5} \right|}{dt}=f5=v_{25}\cdot\left| A_{2} \right|-u_{25}\cdot\left| A_{5} \right|+v_{35}\cdot\left| A_{3} \right|-u_{35}\cdot\left| A_{5} \right|-v_{56}\cdot\left| A_{5} \right|+u_{56}\cdot\left| A_{6} \right|$$

$$\frac{d\left| A_{6} \right|}{dt}=f6=v_{46}\cdot\left| A_{4} \right|-u_{46}\cdot\left| A_{6} \right|+v_{56}\cdot\left| A_{5} \right|-u_{56}\cdot\left| A_{6} \right|-v_{out}\cdot\left| A_{6} \right|$$

(S9)

$$J1=\left[ \begin{matrix} -100 & 2 & 300 & 0 & 0 & 0 \\ 30 & -10 & 0 & 180 & 2 & 0 \\ 50 & 0 & -1000 & 0 & 2 & 300 \\ 0 & 3 & 0 & -300 & 0 & 0 \\ 0 & 1 & 100 & 0 & -10 & 500 \\ 0 & 0 & 0 & 100 & 4 & -1100 \end{matrix} \right]$$

$$J2=\left[ \begin{matrix} -100 & 10 & 300 & 0 & 0 & 0 \\ 30 & -50 & 0 & 180 & 2 & 0 \\ 50 & 0 & -1000 & 0 & 2 & 300 \\ 0 & 15 & 0 & -300 & 0 & 0 \\ 0 & 1 & 100 & 0 & -10 & 500 \\ 0 & 0 & 0 & 100 & 4 & -1100 \end{matrix} \right]$$

(S10)

To evaluate the new regression loss Jacobian algorithm, we utilize an abstract test model and several published models obtained from the EBI BioModels database [6]. The following models are utilized in this evaluation, using reaction perturbations as described to obtain the two conditions (‘h’ and ‘d’):

1. Abstract test model with six components.
2. Model of the upper glycolysis pathway from Klipp et al. [7]: Similar to previous work in Kugler et al. [5], in order to mimic a second network condition, we introduced a twofold increase of the phosphorylation rate parameter k4 of reaction $R4: Fruc6P+ATP\to Fruc1, 6P_{2}+ADP$ from its nominal value$k_{4}^{h}=1$to$k_{4}^{d}=2$. Figure 2a shows the exact differential Jacobian matrix $D\boldsymbol{J}$ between these two conditions at the steady state.
3. Model of the EGFR/ERK signaling pathway in Orton et al. [8]: This work studied the mutation of SOS feedback reactions. The differential Jacobian matrix of the wild type and mutation models is shown in Figure 3a. These two models are also used as evaluation models in [5].
4. Mathematical model of carbohydrate energy metabolism [9]: We increased the reaction rate parameter in $R2: Pyrute+NADHc\to Lactose+NADc$ five-fold for the second conditional Jacobian matrix. Figure 2c shows the exact differential Jacobian matrix for this model.
5. AMPK-mTOR pathway model [10]: The paper describes a wild type and mTOR knockout model based on time-series experimental data, which represent our two conditional Jacobian models. The exact differential Jacobian matrix is in Figure 3b.
6. Hepatic glucose metabolism model [11]. We applied a two-fold parameter change for the reaction rate of the second reaction R2 to generate the second conditional model. The related differential Jacobian matrix is shown in Figure 3c.
7. Large-scale blood cell metabolism model [12]. We introduced a five-fold increase to several components of the Jacobian matrix directly; the exact differential Jacobian matrix is shown in Figure 3d.

# 4 Evaluation approach

To evaluate the inverse Jacobian approach, similar to previous studies [1, 3-5], we utilize the following two approaches to generate the in-silicon data.

The first approach represents a situation in which the covariance matrix C is calculated from a sufficiently large number of samples. In that case, since the simulated fluctuation matrix $\boldsymbol{D}_{\boldsymbol{real}}=\boldsymbol{J}*C_{simulated}+C_{simulated}*\boldsymbol{J}^{T}$ satisfies Eq. (3), we randomly generate $\boldsymbol{D}_{\boldsymbol{real}}=\boldsymbol{D}_{\boldsymbol{theoretical}}+\varepsilon_{D}*rand(\boldsymbol{D})$ near the theoretical fluctuation matrix $\boldsymbol{D}_{\boldsymbol{theoretical}}$ for the two conditions ${\boldsymbol{D}\boldsymbol{1=D}}_{\boldsymbol{health}}$ and ${\boldsymbol{D}\boldsymbol{2=D}}_{\boldsymbol{disease}}$. Here we randomly generate a fluctuation matrix as $\varepsilon_{D}*rand (\boldsymbol{D})$where $rand(\boldsymbol{D})$ is a random $n\times n$ matrix with components bound between -1 and 1$.$ Then we calculate the resulting $C_{1}=C_{health}$ and $C_{2}=C_{disease}$ with the Lyapunov equation. The aim of this evaluation is to verify the algorithm feasibility when the sample size is enough. As shown in figure S2, we tested the average $\varepsilon_{D}$ for different SDE simulation replicates using the test model. Here we enhance the last row of the original Jacobian matrix for different folds and generate several modified test model (Jacobian matrix condition number 100-100000). As in figure S2, the necessary sample size for $\varepsilon_{D}=0.5$ will be over ${10}^{4}$ for a Jacobian with a large matrix condition number. This evaluation approach will save a lot of time for the stochastic differential equations (SDE) simulation.

In the second approach, we apply stochastic differential equations (SDE) simulation to the model, obtaining a small number of samples (100 to 1000). This approach will evaluate the method’s accuracy for large-scale experiments (as some large-scale metabolomics studies will have on the order of ${10}^{2\sim3}$ samples). Note that SDE simulation is hardly feasible for large sample numbers (e.g., 10000 samples for the blood cell metabolism model would take several weeks on a standard desktop computer as shown in Figure S3). We use SDE simulation to generate in-silicon data of the test model. For each model, we added the stochastic Gaussian noise perturbations to components at each time step [13], which corresponds to the nominal ***D*** matrix being a diagonal matrix. Moreover, we repeated the computation with covariance computed from 100 and 1000 samples to evaluate the effect of sample size on the results. The SDE solving method is the second-third order Runge-Kutta implicit method.


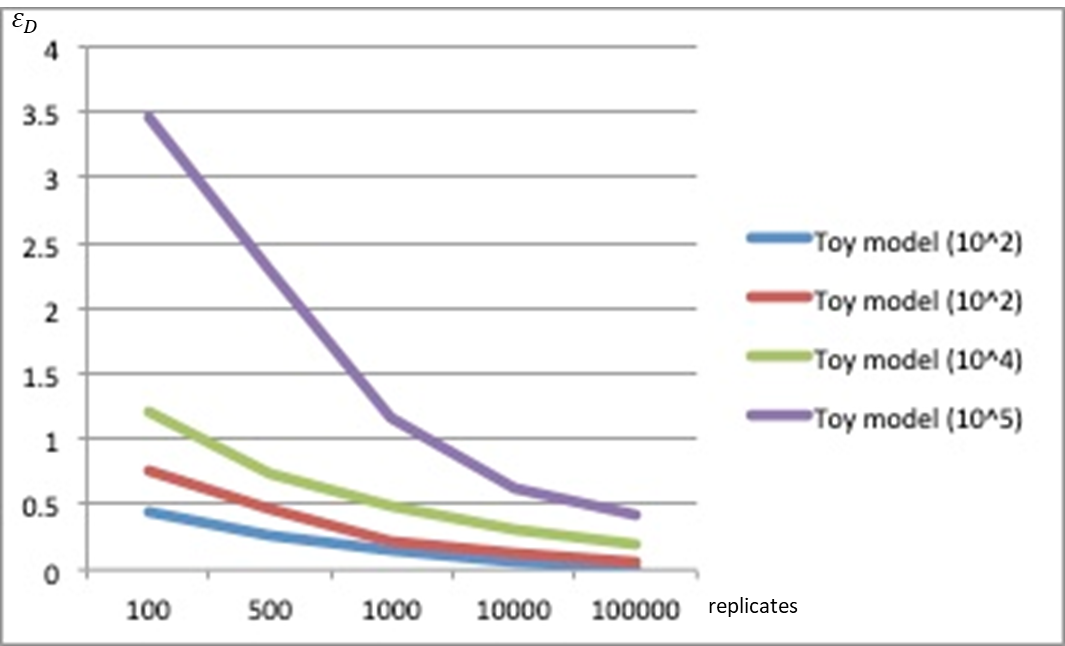


Figure S2. The average $\varepsilon_{D}$of different sized replicates with SDE simulation. The test is carried out using the test model, with different Jacobian matrix number scale (100-100000).


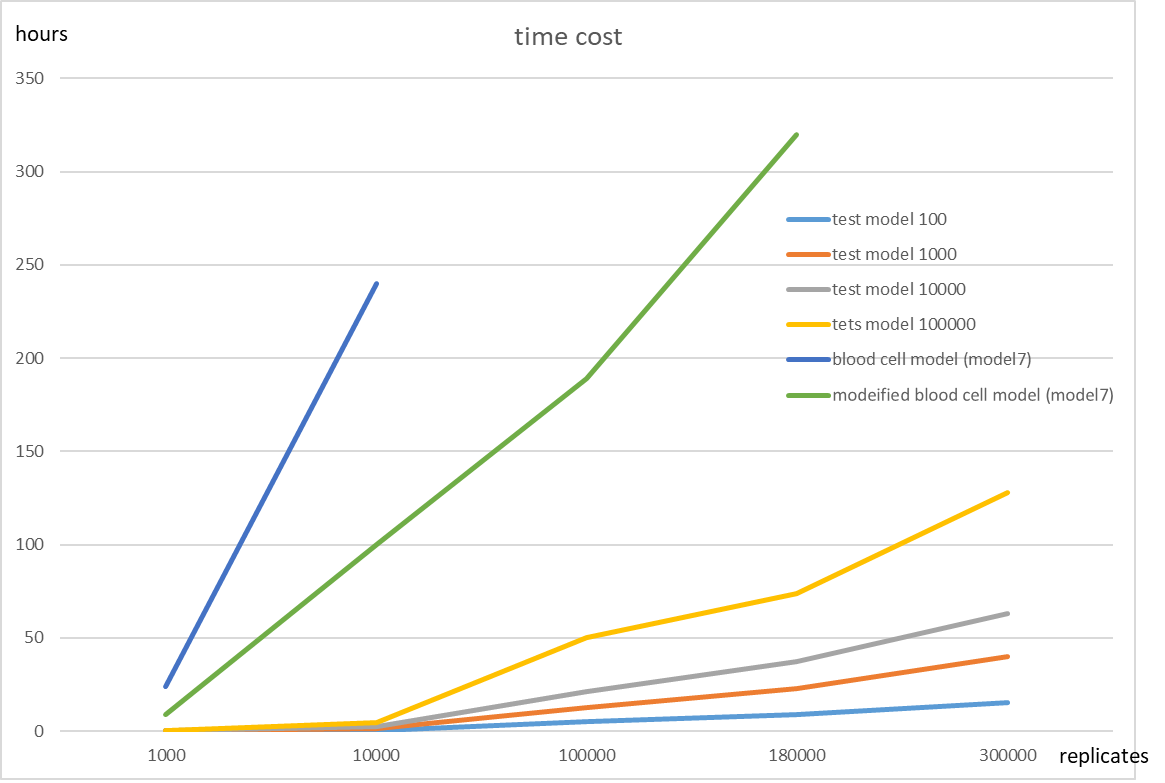


Figure S3. The time cost for the SDE simulation for different replicates with different models. Here the modified blood cell model has a much smaller Jacobian matrix condition number (~100000), which is generated through randomly changing the Jacobian components in the original model.

# 5 Effect of the network reduction on the Jacobian reconstruction

The first step in COVRECON is based on a network reduction from a genome-scale database to the metabolites present in the considered dataset. To test the effect of this network reduction on the reliability of the Jacobian reconstruction, we evaluate these steps using with models 4, 6 and 7 introduced before (carbohydrate energy metabolism model, hepatic glucose metabolism model and the blood cell metabolism model).

For the carbohydrate energy metabolism model, we first randomly added six intermediate products to enlarge the original model from size 12 to 18, and then computed a Jacobian reconstruction based on a reduction from the enlarged model. With the other two models, we chose 14-17 core metabolites for which to build reduced models. For each of the three models, we first generated the simulated covariance matrices $C_{h} \mathrm{and}$ $C_{d}$ of the larger model for both conditions ‘d’ and ‘h’ using the Lyapunov equation as described in Section 2.3.3 with $\varepsilon_{D}=0.5$. Then we use the Sim-network reduction strategy as described in Section 2.2 to generate the Jacobian matrix structure to be used for the inverse Jacobian. Using the covariance matrix together with the Jacobian matrix structure for the components that are kept in the reduced network, we run the new inverse Jacobian algorithm. The reconstruction results are summarized in Figure S4. For the first model (carbohydrate energy metabolism), all relevant components of the differential Jacobian in the full network are detected in the lower-dimensional reconstruction (Fig. S4a). As for the other two, more complex, models, most of the large components of the differential Jacobian in the original network will also be detected by the reconstruction with the reduced network structure, however, a few elements of the differential Jacobian which have large values in the original network are not detected in the reduced network. Some of these false negatives can be attributed to actual information loss due to the network reduction (e.g. the influence from udpglc to glyglc in hepatic glucose metabolism model, Figure S4b; the influence from NAD to Pyr & Lac, Figure S4c), others result from limitations in the reconstruction itself not stemming from the network reduction. In fact, in the blood cell metabolism model (Figure S4c), the reconstruction with the reduced network structure detects the same components as when using the original model structure directly (Fig. S4d).


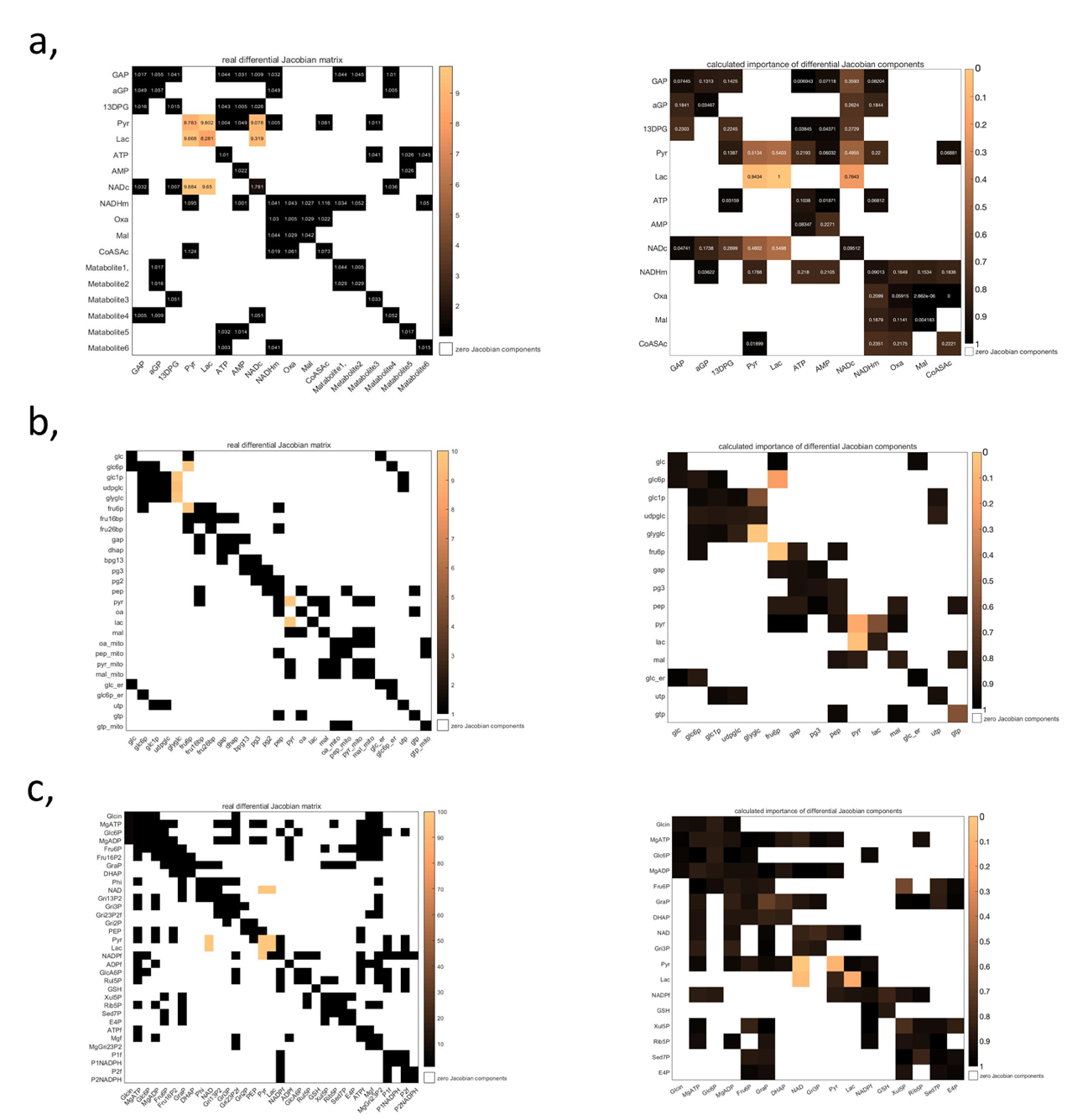


FigureS4. Network reduction test for three models: carbohydrate energy metabolism model (a), hepatic glucose metabolism model (b) and the blood cell metabolism model (c). The test is carried out through the first evaluation approach while setting $\varepsilon_{D}=0.5$.

In conclusion, this section verifies the feasibility of the first step (Sim-network) in the COVRECON approach. Even when using the reduced network structure for the Jacobian reconstruction, the algorithm is able to detect most of the relevant interactions in the original network.

# 6 Sim-Network Matlab Interface and default settings

This whole workflow COVRECON has been implemented in a Matlab interface, and available in … . The general instruction of this interface is presented in Figure S5, and the default settings are shown in Table S1. A toolbox manual is presented in Supplemental Material S3.

**A,**

**
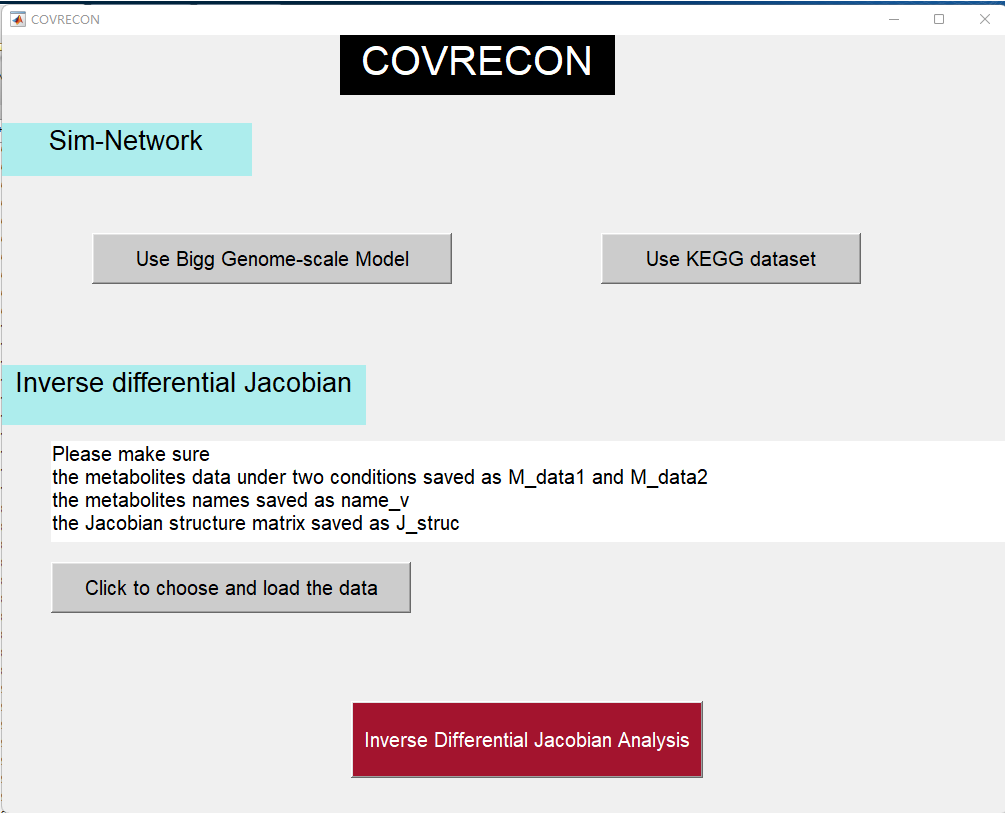
**

**B,**

**
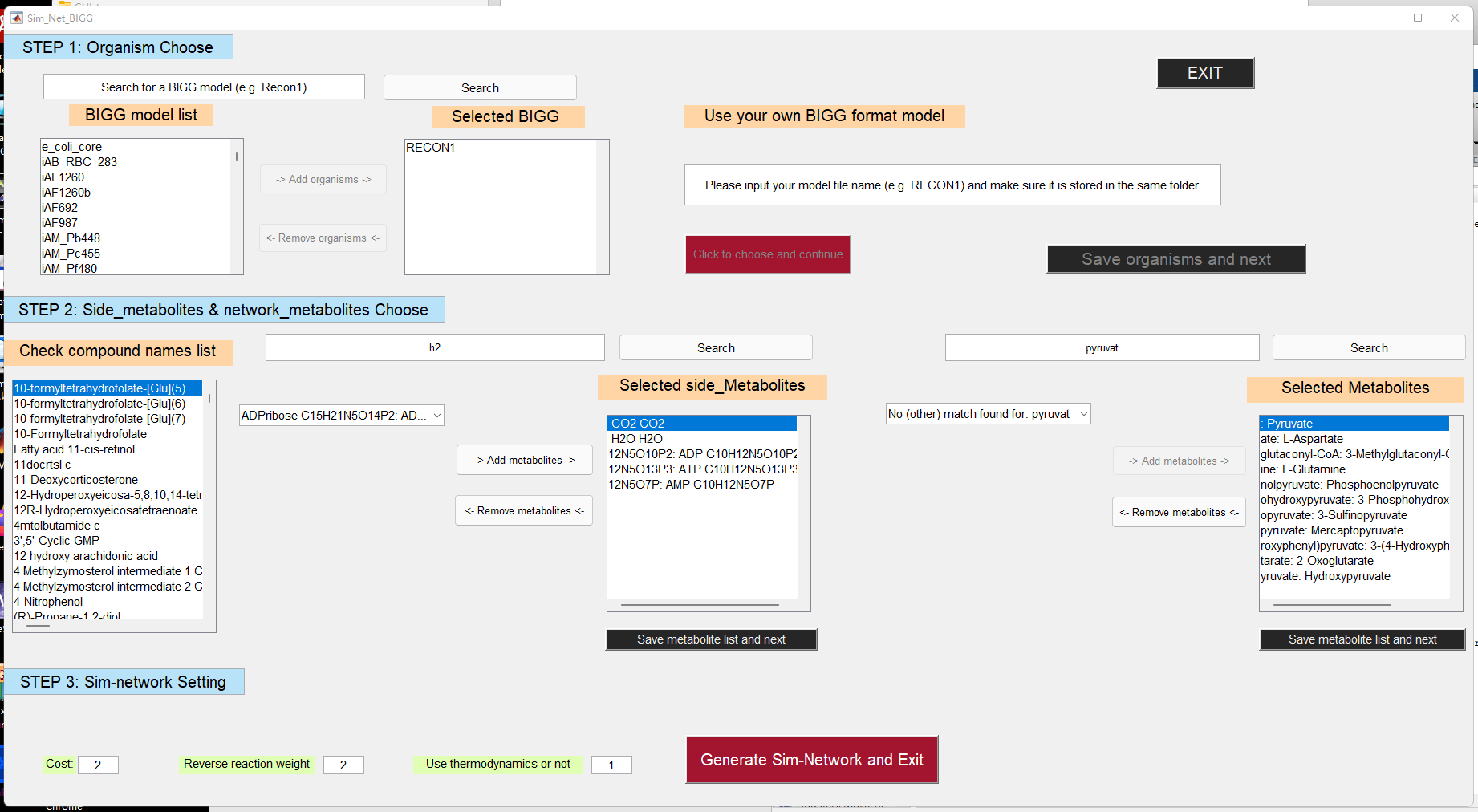
**

Figure S5. The Matlab interface for COVRECON workflow. Figure S5A is the main interface of COVRECON. It includes parts: Sim-Network and Inverse Jacobian. One can choose to use Bigg model or KEGG dataset in Sim-Network, which lead to the secondary interface as in Figure S5B. The Sim-Network tool has three steps: first choose a dataset; then search and select side-metabolites and measured metabolites in one’s metabolomics dataset; finally set the network constructing settings.

Table S1. Sim-Network default settings

# 7 Supplementary tables and figures

## Supplementary Figure 1.

Comparison between differential Jacobian matrixes and differential correlation matrixes using four models 1, 4, 6 and 7 as in Section 2.5, with perturbation level $\varepsilon_{D}=0.4$.


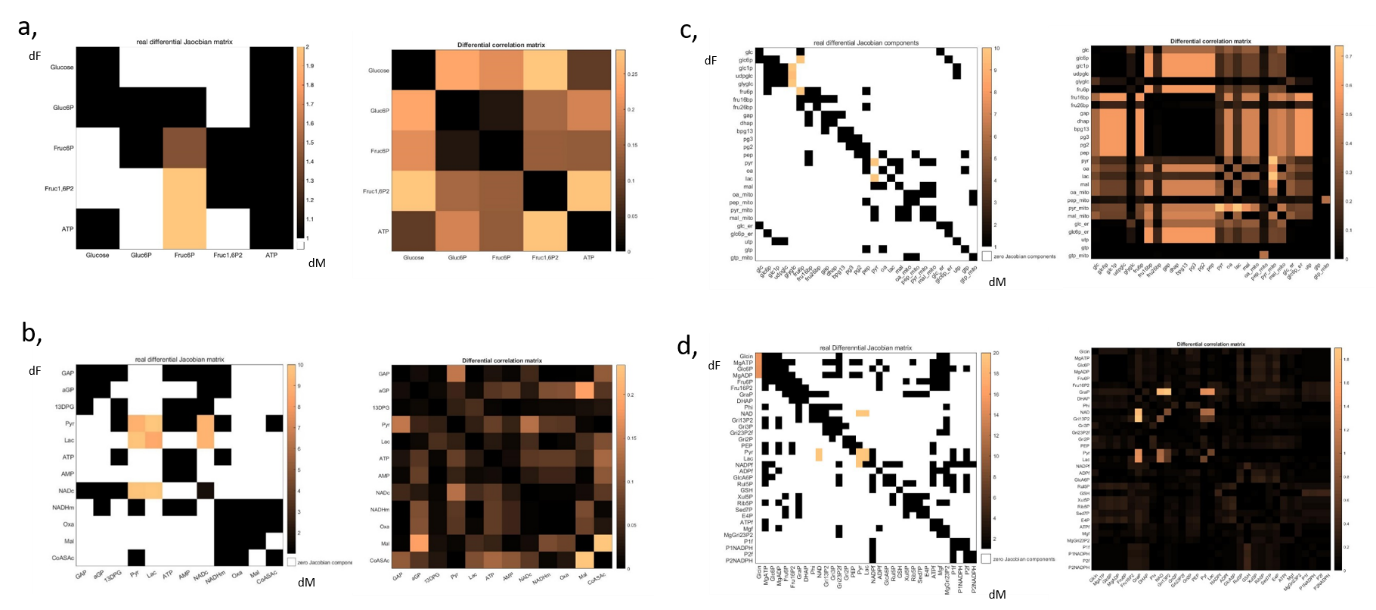


## Supplementary Table 1. Evaluation of three inverse differential Jacobian approaches with the test model.

The cost is the L-p optimization loss; the target cost is the loss with the real $b_{h} \mathrm{and} b_{d}$. As a further analysis, we introduced a tenfold change to three randomly chosen components in the Jacobian matrix and generated all 1140 inverse tasks with different ‘d’ conditional Jacobian matrix. The new regression loss Jacobian algorithm is able to find the exact three components for 335/1140 tasks, 2 components for 605/1140 tasks and can only find zero or one correct component for 200/1140 tasks, respectively.

## Supplementary Figure 2

The actual matrices of the inverse differential Jacobian analysis results with both L-P optimization and the new regression loss Jacobian algorithm for all evaluation models in section 2.5. In each sub-graph, the left subplot gives the exact differential Jacobian matrix; the middle subplot shows the calculated differential Jacobian matrix through L-p optimization approach; the right subplot presents the regression loss Jacobian algorithm results, which is the best regression loss matrix $R^{*}=[r^{*}(I)]$ as in Supplemental material Eq.$\left( S4 \right)$&$({S4}^{*})$, with component values scaled to the interval (0,1). Where the regression loss$r^{*}(I)$ at the Jacobian component $J_{ij}$is the best regression loss with setting component $I=J_{ij}$as the only differential Jacobian component.


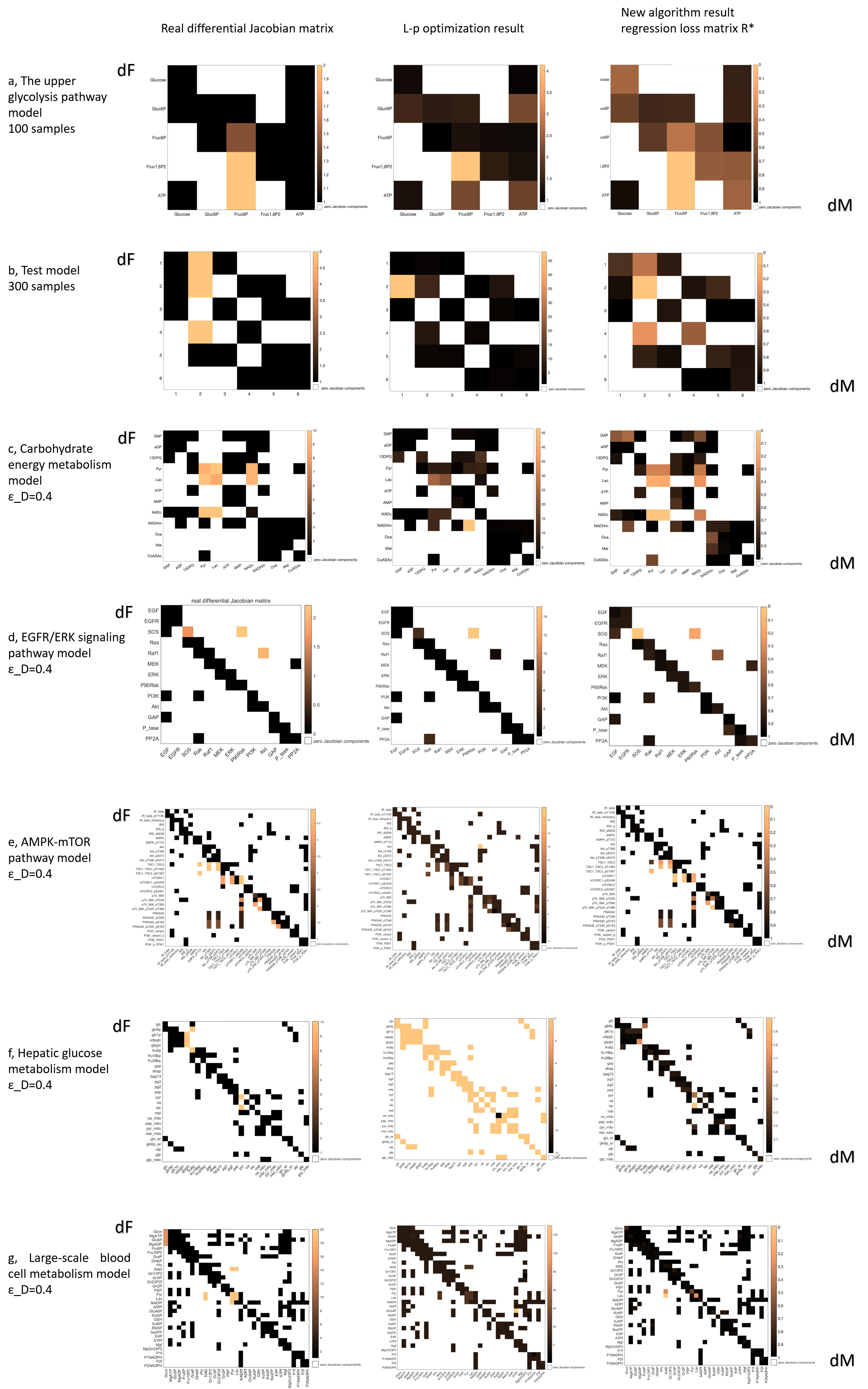


## Supplementary Figure 3.

The scatter plot between the real differential Jacobian components values (scaled to 0-1) and the calculated $r\left( i,j \right)$ in our regression loss Jacobian algorithm for all models results in Figure 2. Here, we set 0.5 as the threshold between positive and negative values.


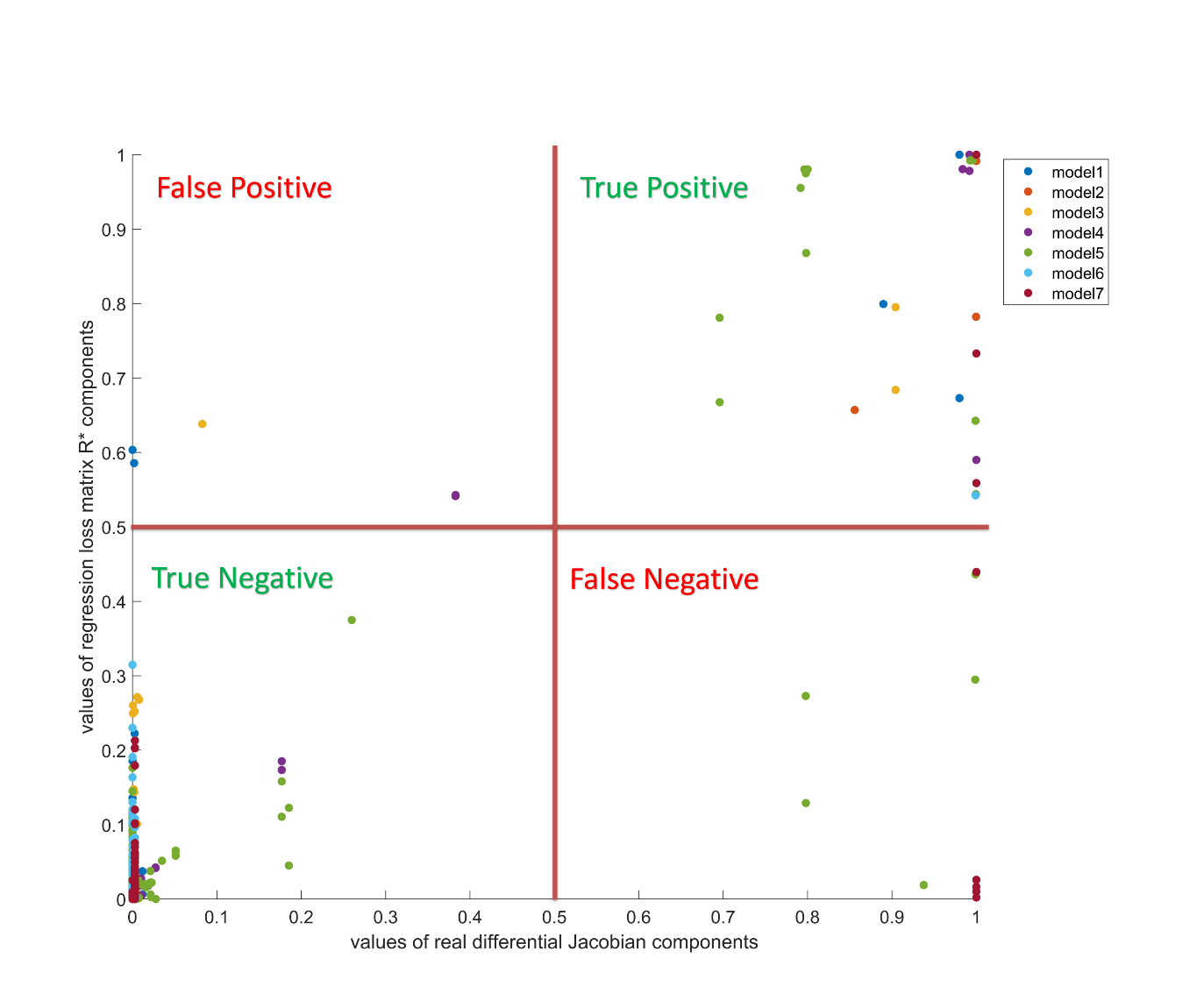


Supplementary Figure 4. The regression loss Jacobian algorithm results for the first four evaluation models 1, 3, 4 and 5 with the covariance matrixes generated from SDE simulation for 1000 and 100 samples, respectively.


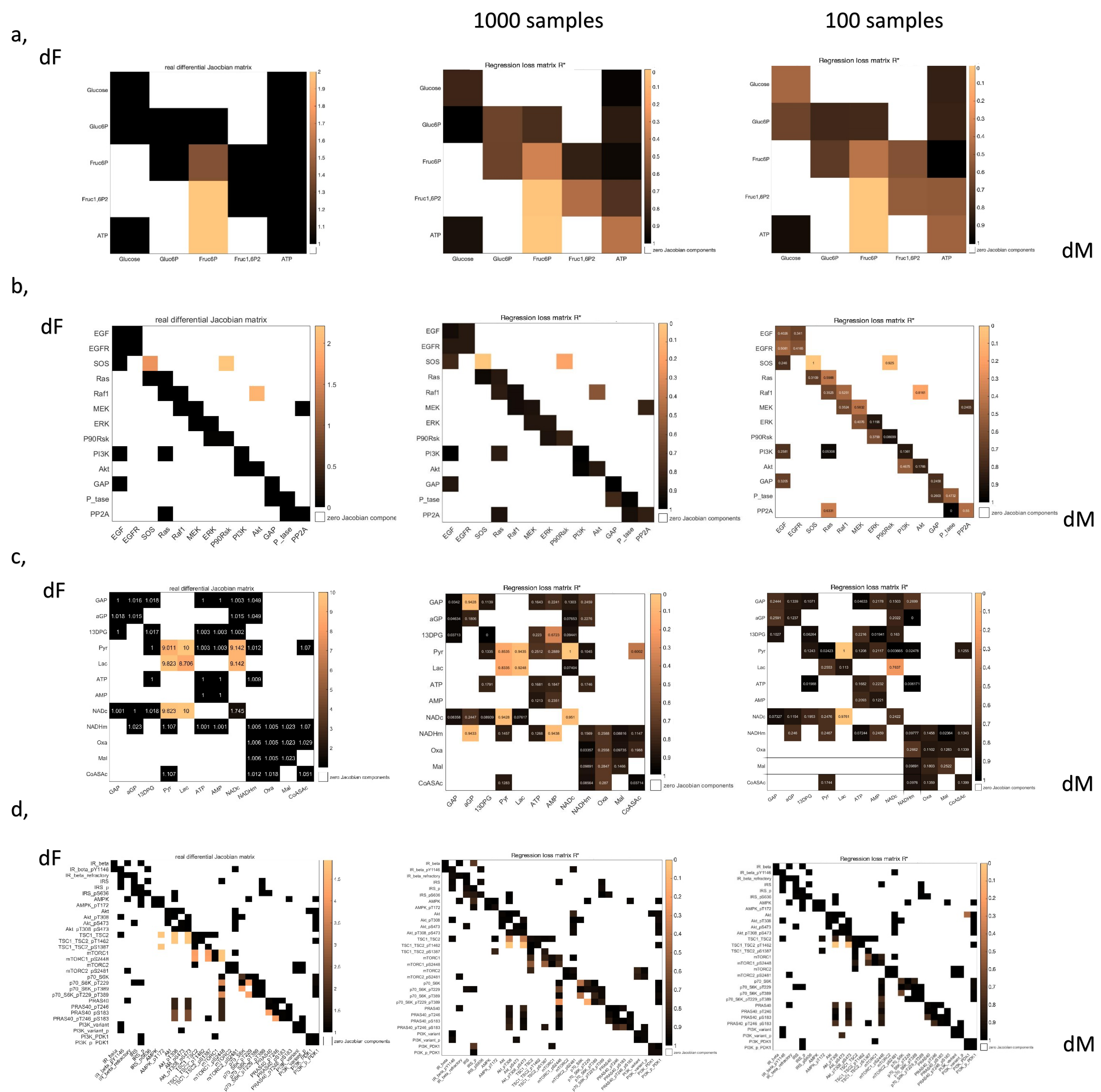


Supplementary Figure 5

The actual matrices (in place of the circular interaction plots) for inverse Jacobian analysis using the models hepatic glucose metabolism model (a,) and large-scale blood cell metabolism model (b,). Regression loss matrix $R^{*}$determined from COVRECON (right) and the exact differential Jacobian matrixes (left).


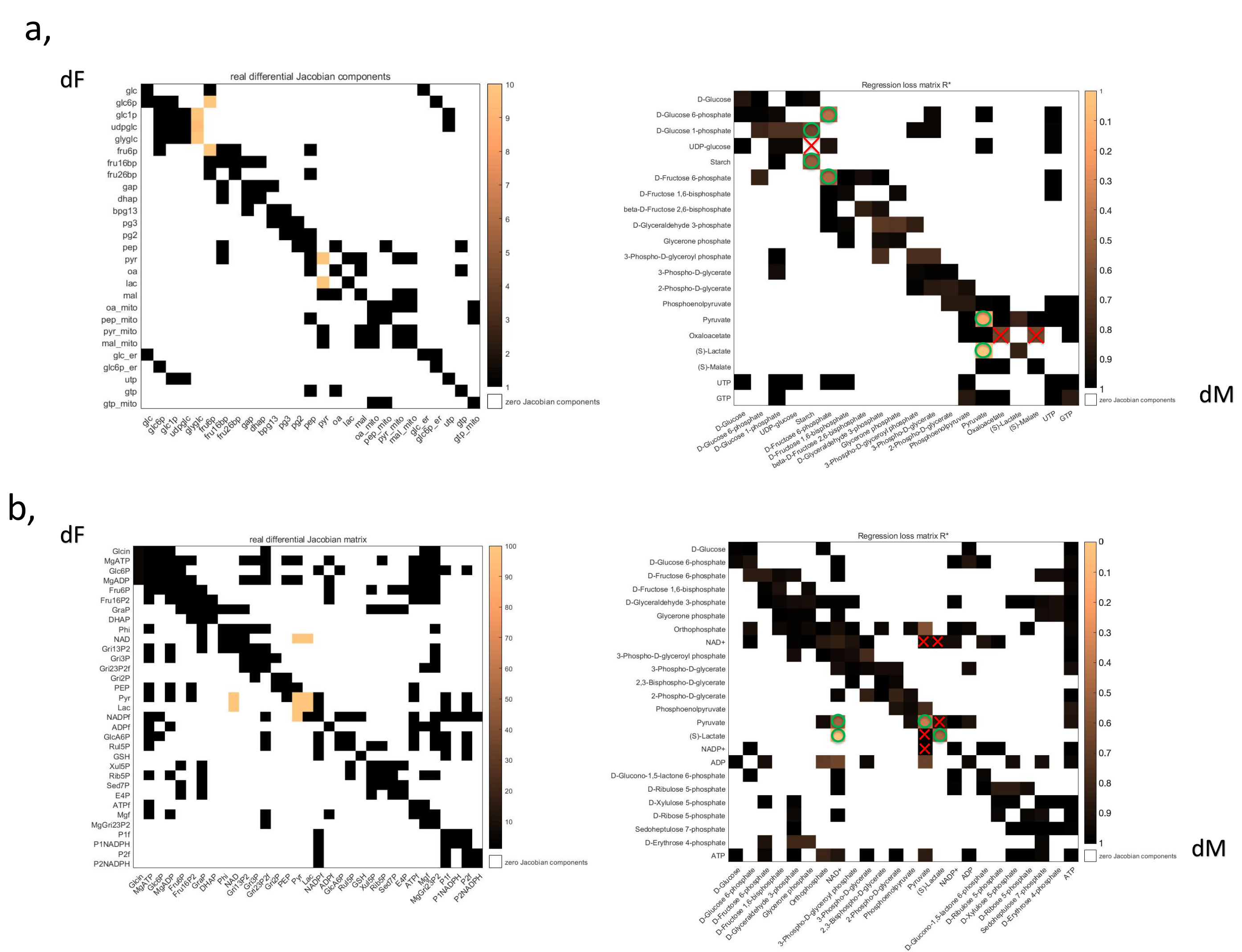


Supplementary Figure 6.

Inverse differential Jacobian analysis for hepatic glucose metabolism model (a,) and large-scale blood cell metabolism model (b,) using Sim-network with a different setting: cost threshold 1, reverse reaction weight 1, no thermodynamics related. From top to bottom, the first subplots are the real differential metabolic interaction networks; the second subplots present the reconstructed Jacobian matrix (color codes refer to Fig. 3); the resulting regression loss matrix $R^{*}$and differential metabolic interaction networks are presented on the bottom two subplots, respectively.


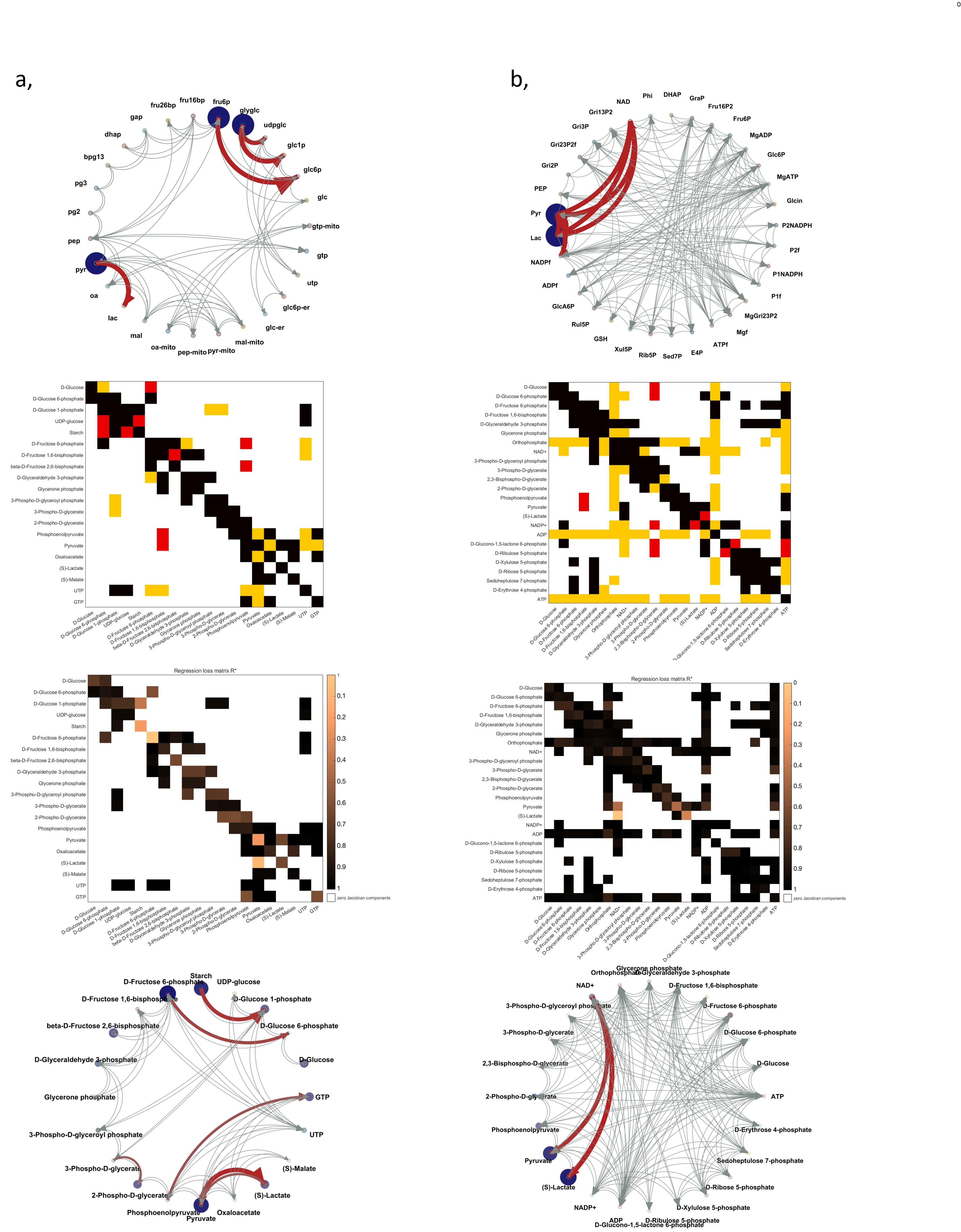


Supplementary Figure 7

The actual matrix plot of the regression loss matrix R* (refer to Fig. 5).


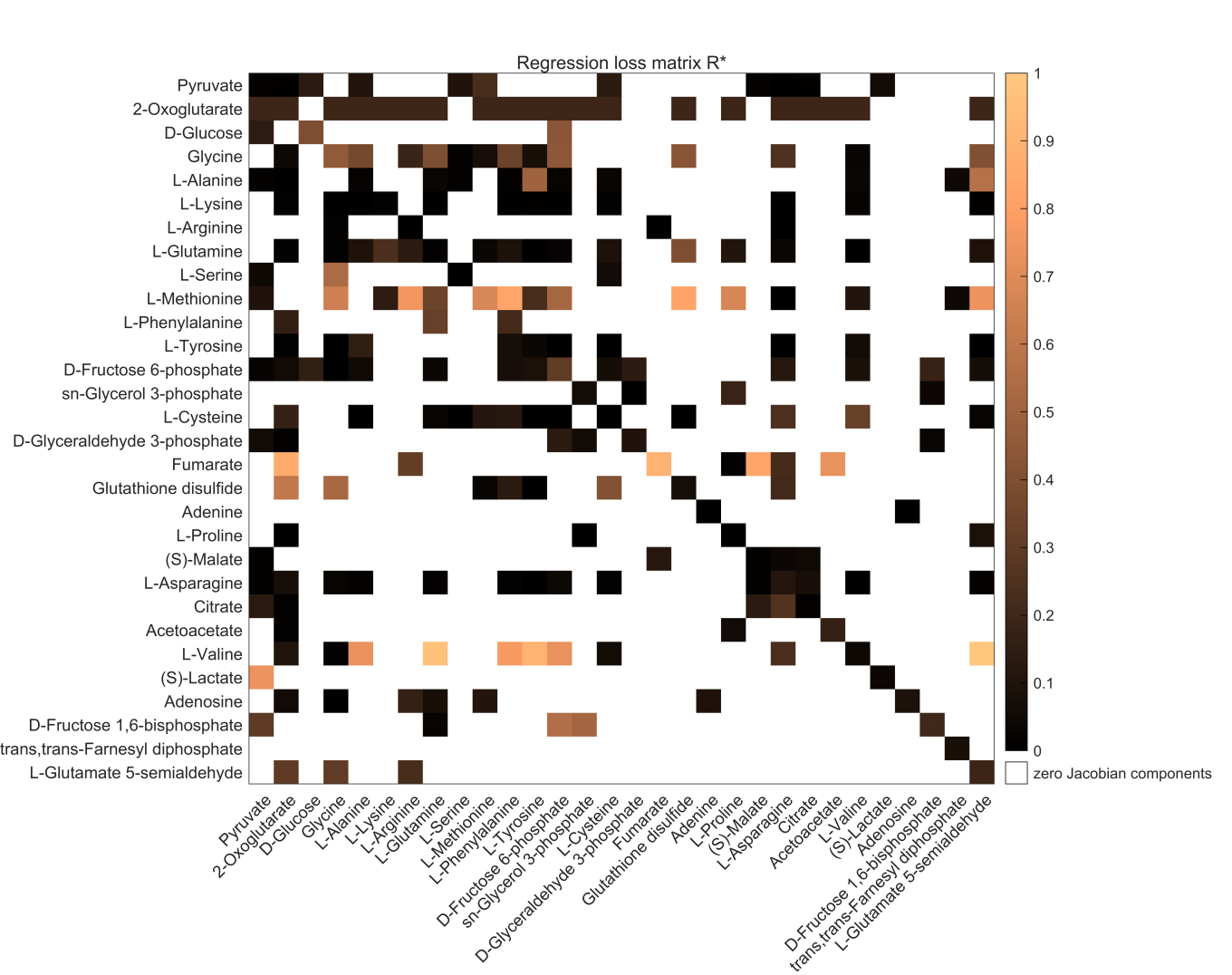


Supplementary Figure 8

The t-test results of the transcriptomic profile for all interactions with highlighted value above 0.5.


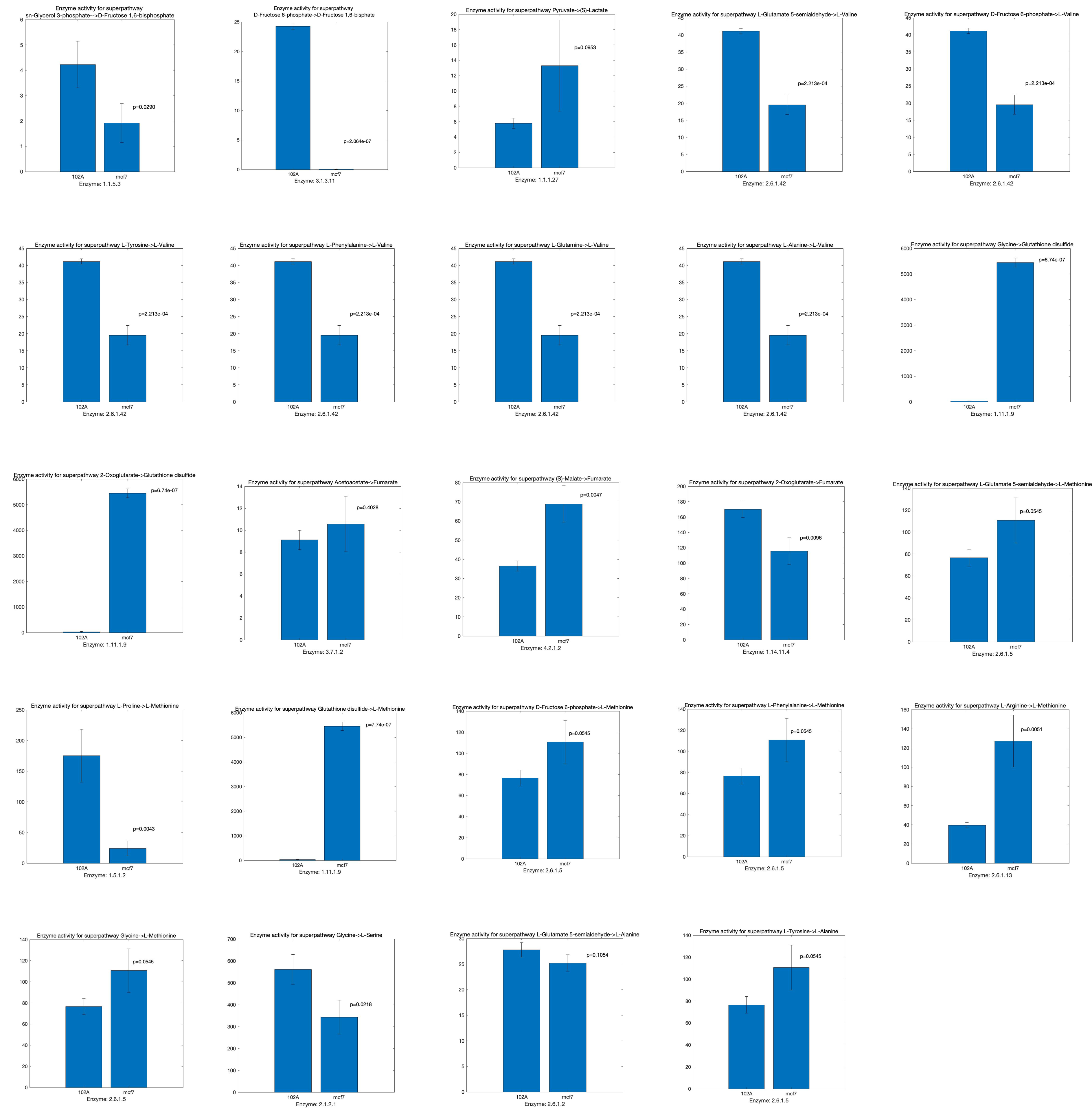


**Reference**

1. Sun, X., B. Länger, and W. Weckwerth, *Challenges of inversely estimating jacobian from metabolomics data.* Frontiers in bioengineering and biotechnology, 2015. **3**: p. 188.

2. Paige, C.C. and M.A. Saunders, *LSQR: An algorithm for sparse linear equations and sparse least squares.* ACM Transactions on Mathematical Software (TOMS), 1982. **8**(1): p. 43-71.

3. Sun, X. and W. Weckwerth, *COVAIN: a toolbox for uni-and multivariate statistics, time-series and correlation network analysis and inverse estimation of the differential Jacobian from metabolomics covariance data.* Metabolomics, 2012. **8**(1): p. 81-93.

4. Nägele, T., et al., *Solving the differential biochemical Jacobian from metabolomics covariance data.* PloS one, 2014. **9**(4): p. e92299.

5. Kügler, P. and W. Yang, *Identification of alterations in the Jacobian of biochemical reaction networks from steady state covariance data at two conditions.* Journal of Mathematical Biology, 2014. **68**(7): p. 1757-1783.

6. Malik-Sheriff, R.S., et al., *BioModels—15 years of sharing computational models in life science.* Nucleic acids research, 2020. **48**(D1): p. D407-D415.

7. Klipp, E., et al., *Systems biology: a textbook*. 2016: John Wiley & Sons.

8. Orton, R.J., et al., *Computational modelling of cancerous mutations in the EGFR/ERK signalling pathway.* BMC systems biology, 2009. **3**(1): p. 1-17.

9. Nazaret, C. and J.-P. Mazat, *An old paper revisited:“A mathematical model of carbohydrate energy metabolism. Interaction between glycolysis, the Krebs cycle and the H-transporting shuttles at varying ATPases load” by VV Dynnik, R. Heinrich and EE Sel’kov.* Journal of theoretical biology, 2008. **252**(3): p. 520-529.

10. Dalle Pezze, P., et al., *A systems study reveals concurrent activation of AMPK and mTOR by amino acids.* Nature communications, 2016. **7**(1): p. 1-19.

11. Bulik, S., H.-G. Holzhütter, and N. Berndt, *The relative importance of kinetic mechanisms and variable enzyme abundances for the regulation of hepatic glucose metabolism–insights from mathematical modeling.* BMC biology, 2016. **14**(1): p. 1-22.

12. Holzhütter, H.G., *The principle of flux minimization and its application to estimate stationary fluxes in metabolic networks.* European journal of biochemistry, 2004. **271**(14): p. 2905-2922.

13. Higham, D.J., *Modeling and simulating chemical reactions.* SIAM review, 2008. **50**(2): p. 347-368.
